# DNA Barcoding and Chemical Profile Using UHPLC, GC-MS and LC-MS/QTOF of *Mitragyna speciosa* Variation and Allied Species for Quality Control of Kratom Materials

**DOI:** 10.3390/plants15071003

**Published:** 2026-03-25

**Authors:** Phunsuk Anantaworasakul, Warunya Arunotayanun, Siripat Chaichit, Suthiwat Khamnuan, Chatchai Ngernsaengsaruay, Chuda Chittasupho, Nisa Leksungnoen, Mingkwan Na Takuathung, Ruedeemars Yubolphan, Apisada Jiso, Tachpon Techarang, Aekkhaluck Intharuksa

**Affiliations:** 1 Regional Medical Sciences Center 2 Phitsanulok, Phitsanulok 65000, Thailand; phunsuk.a@dmsc.mail.go.th; 2 Kanchanabhishek Institute of Medical and Public Health Technology, Faculty of Public Health and Allied Health Science, Praboromarajchanok Institute, Nonthaburi 11150, Thailand; warunya@kmpht.ac.th; 3 Department of Pharmaceutical Sciences, Faculty of Pharmacy, Chiang Mai University, Chiang Mai 50200, Thailand; siripat.chaichit@cmu.ac.th (S.C.); chuda.c@cmu.ac.th (C.C.); apisada.j@cmu.ac.th (A.J.); 4 Faculty of Pharmacy, Western University, Pathum Thani 12150, Thailand; suthiwat.khamnuan@gmail.com; 5 Department of Botany, Faculty of Science, Kasetsart University, Chatuchak, Bangkok 10900, Thailand; fsciccn@ku.ac.th (C.N.); ffornsl@ku.ac.th (N.L.); 6 Department of Pharmacology, Faculty of Medicine, Chiang Mai University, Chiang Mai 50200, Thailand; mingkwan.n@cmu.ac.th (M.N.T.); ruedeemars.yub@cmu.ac.th (R.Y.); 7 Clinical Research Center for Food and Herbal Product Trials and Development (CR-FAH), Faculty of Medicine, Chiang Mai University, Chiang Mai 50200, Thailand; 8 Department of Tropical Pathology, Faculty of Tropical Medicine, Mahidol University, Bangkok 10400, Thailand; tachpon.tec@mahidol.ac.th

**Keywords:** ketum, quality control, narcotics, herbal medicine, mitragynine

## Abstract

Kratom (*Mitragyna speciosa* Korth.) has gained increasing global attention due to its traditional use, psychoactive properties, and emerging therapeutic potential; however, concerns regarding adulteration, substitution, and inconsistent quality of commercial products necessitate robust authentication strategies. This study aimed to integrate DNA barcoding and comprehensive chemical profiling to authenticate kratom variants and discriminate them from closely allied *Mitragyna* species for quality control and forensic applications. Nine DNA barcoding regions were analyzed, alongside chemical characterization using UHPLC, GC–MS, and LC–MS/QTOF. Among the tested loci, the internal transcribed spacer (ITS) and ITS2 regions exhibited the highest interspecific variation and effectively distinguished kratom from allied species. UHPLC and GC–MS analyses confirmed that mitragynine was exclusively detected in kratom variants, with Kan Khiao exhibiting the highest content (94.33 ± 0.14 mg/g) when quantified against the mitragynine standard using UHPLC analysis. LC–MS/QTOF profiling revealed an alkaloid-rich chemotype in kratom dominated by mitragynine and 7-hydroxymitragynine, whereas *M. diversifolia*, *M. hirsuta*, and *M. rotundifolia* showed distinct profiles enriched in phenolic acids and flavonoid glycosides. Multivariate analyses further identified procyanidin B1, datiscetin-3-O-rutinoside, mitragynine, and 7-hydroxymitragynine as key discriminatory markers. Overall, the combined molecular and chemical workflow provides a robust framework for kratom authentication, supporting regulatory monitoring, quality assurance, and forensic identification of kratom materials.

## 1. Introduction

*Mitragyna speciosa* Korth., of the family Rubiaceae, is a tropical tree indigenous to Southeast Asia—particularly Thailand and Malaysia—and is also found in Indonesia, the Philippines and Myanmar; locally, it is known as “kratom,” “ketum,” or “biak-biak.” Its leaves have long been used in traditional practice to manage pain, hypertension, fever, cough, fatigue and diarrhea. In daily use, local people ingest raw leaves or consume brewed or steeped decoctions from the leaves for stimulant effects and to alleviate fatigue and musculoskeletal discomfort, particularly among rural laborers [1,2].

**Figure 1:**
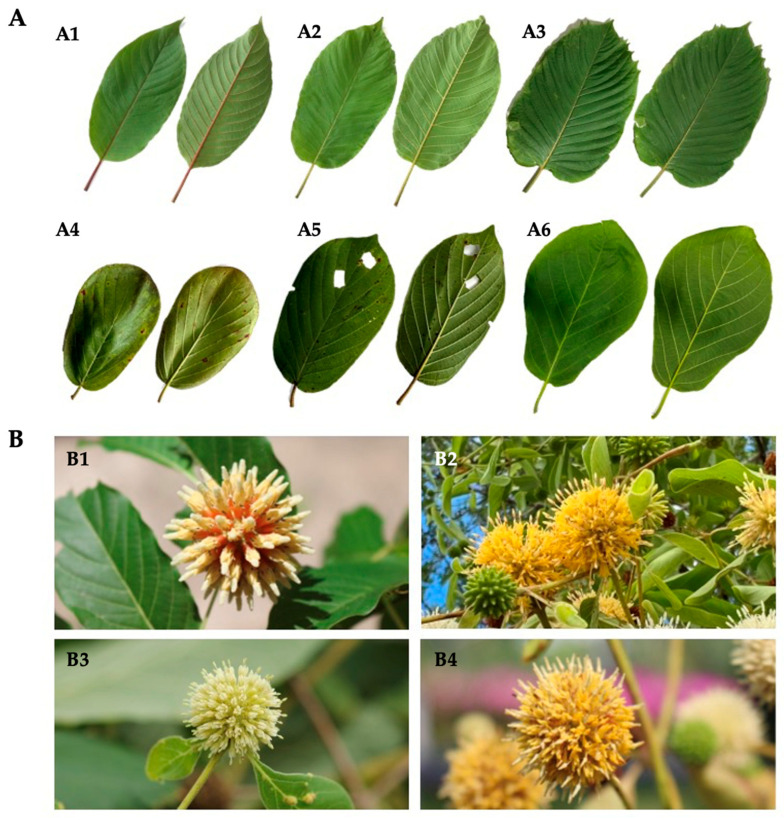
*Mitragyna* spp. in Thailand. (**A**) Leaves: *M. speciosa* (kratom)—Kan Daeng ((**A1**), red vein), Kan Khiao ((**A2**), green vein), Hang Kang ((**A3**), serrated-margin type); allied species—*M. diversifolia* (**A4**), *M. hirsuta* (**A5**), *M. rotundifolia* (**A6**). (**B**) Flowers: *M. speciosa* (**B1**), *M. diversifolia* (**B2**), *M. hirsuta* (**B3**), and *M. rotundifolia* (**B4**).

*M. speciosa* comprises numerous strains that differ in chemistry and effects. Strains are commonly classified by leaf shape, vein color or growth pattern (green, red, white); these categories correlate with distinct alkaloid profiles and reported pharmacological activities [3,4,5]. Beyond *M. speciosa*, the genus *Mitragyna* comprises about ten additional species distributed across tropical Asia and Africa; Asian species include *M. diversifolia* (Wall. ex G. Don) Havil., *M. hirsuta* Havil., *M. parvifolia* (Roxb.) Korth., *M. rotundifolia* (Roxb.) Kuntze, and *M. tubulosa* (Arn.) K. Schum. (occurring in Southeast Asia, China/Indochina, Bangladesh, and India), whereas African taxa include *M. ciliata* Aubrév. & Pellegr., *M. inermis* (Willd.) Kuntze, *M. rubrostipulata* (K.Schum.) Havil., and *M. stipulosa* (DC.) Kuntze [2,6]. Notably, several countries in Southeast Asia host multiple *Mitragyna* species; for example, Thailand is a rich source, including *M. speciosa* (kratom; Kan Khiao—green vein, Kan Daeng—red vein, Hang Kang—serrated-margin type) and allied species such as *M. diversifolia* (Kra thum na), *M. hirsuta* (Kra thum khok), and *M. rotundifolia* (Kra thum noen) (Figure 1). Green-vein kratom usually provides moderately stimulatory and analgesic effects, while red-vein kratom offers sedative and analgesic effects [5]. Ethnomedicinal use is widespread across the genus. Several Asian *Mitragyna* species are employed as kratom substitutes. Documented uses include *M. parvifolia* for pain, swelling, and diabetes; *M. hirsuta* to promote lactation; *M. rotundifolia* for fracture-related pain and swelling; and *M. diversifolia* for antidiarrheal effects [2]. *Mitragyna* leaves are employed internally and externally for diverse complaints with overlap among species (e.g., gastrointestinal support, fatigue, dermatologic uses), but the literature most consistently documents *M. speciosa* as the only opium substitute and remedy for addiction. Other *Mitragyna* species are chiefly reported for pain relief via topical preparations, with additional uses including treatment of fever, skin infections, and mild anxiety [6].

Phytochemical studies of *Mitragyna* leaves have shown that alkaloids are the principal constituents, alongside other secondary metabolites, such as flavonoids, polyphenols, triterpenoids, monoterpenes, and secoiridoids. Within the genus, *M. speciosa* has been studied most extensively and is found to contain appreciable amounts of pharmacologically active corynanthe-type monoterpene indole alkaloids (MIAs) [2,6]. Mitragynine is the major alkaloid of kratom, constituting up to approximately 60% (*w*/*w*) of the total alkaloids in some materials [7], alongside 7-hydroxymitragynine, speciociliatine, speciogynine, mitraphylline, and paynantheine, with proportions varying by geographic origin [1,6]. Mitragynine and related analogs show affinity for μ- and κ-opioid receptors, contributing to analgesic, opioid-like effects and supporting proposed mechanisms relevant to opioid-withdrawal therapy. Although a trace constituent, 7-hydroxymitragynine interestingly exhibits potent antinociceptive activity [8]. Anti-inflammatory actions have also been reported, including suppression of PGE_2_ production via the COX-2 pathway [9]. Moreover, the compound interacts with additional targets, including adrenergic and serotonergic receptors and neuronal Ca^2+^ channels in the central nervous system, contributing to reported neuropharmacological effects such as increased physical endurance, sedation, stimulation, antidepressant and anxiolytic actions, and potential anti-obesity effects [10,11]. These activities may offer therapeutic benefits (e.g., management of opioid dependence and neurodegeneration) but also carry risks of adverse effects. Beyond the CNS, kratom has been reported to exhibit gastrointestinal effects and antioxidant, antibacterial, and anti-breast cancer activities [10,11].

Owing to activity at opioid receptors, kratom exhibits opioid-like analgesia and has been used as an opium or morphine substitute in the management of drug dependence. In recent years, kratom has attracted increased attention for its psychoactive effects and has become one of the most prevalent “legal highs” on the market [12]. It is sold in various forms, including dried powders, capsules, extracts, and ready-to-drink formulations, and used recreationally in many countries [1,6,13]. Kratom’s legal status remains heterogeneous worldwide, with policies toward *M. speciosa* and its principal alkaloids (mitragynine, 7-hydroxymitragynine) ranging from legal to partially legal to prohibited. In Southeast Asia, Malaysia, Myanmar, and Singapore prohibit consumption, possession, and trade (Malaysia under the Poisons Act 1952), whereas Indonesia lacks explicit, detailed controls despite being a major exporter [14,15]. Thailand removed *M. speciosa* from the Narcotics Act in 2021 and enacted the Kratom Plant Act in 2022, legalizing cultivation, leaf harvesting, and traditional consumption (chewing/decoction) while restricting import/export, sales to minors and pregnant or nursing women, and prescribing labeling/advertising requirements [16]. In Europe, several countries (e.g., Denmark, France, Portugal, Poland, Turkey, and Italy) regulate kratom and/or its alkaloids, whereas the Netherlands and Germany permit trade or possession within defined regulatory frameworks [14]. In the United States, legal status varies by jurisdiction at the state, county, and municipal levels; many areas permit sales, whereas others impose restrictions or categorical bans on sale and use [17].

Global consumption of *M. speciosa* (kratom) has risen rapidly, driven by perceived therapeutic and psychoactive effects. There are growing concerns about product purity and inadequate quality control, including reports of adulteration and inconsistent alkaloid profiles arising from cultivation and processing variability [13]. Regulatory disparities across countries have also prompted online marketing of other *Mitragyna* species as “legal alternatives,” with increasing evidence of substitution in products sold as kratom. These issues underscore the need for rigorous authentication and standardized testing across the supply chain [2,6,18]. As macro- and microscopic identification is often insufficient for species-level discrimination, phytochemical profiling and molecular approaches offer more reliable authentication [6]. Robust, multi-technique workflows are therefore required for *M. speciosa* and allied species to establish medicinal chemistry, safety, and toxicology parameters that meet regulatory requirements for quality control of commercial materials and forensic applications. In response to these gaps, the present study aimed to provide DNA barcoding and UHPLC, GC-MS, and LC-MS/QTOF chemical profiling of *M. speciosa* variations and allied species to strengthen authentication, support quality control, forensic application, and inform biological risk assessment.

## 2. Results

### 2.1. Molecular Sequencing Analysis of Kratom Variations and Other Mitragyna Species

DNA barcoding was employed to discriminate kratom (*Mitragyna speciosa*) from closely allied *Mitragyna* species, including *M. diversifolia*, *M. hirsuta*, and *M. rotundifolia* (Table S1). In addition, this approach was used to assess the genetic variation among different kratom cultivars, namely Kan Daeng, Kan Khiao, and Hang Kang (Table S1). A total of eight genetic loci were selected for sequencing analysis, comprising the nuclear ribosomal internal transcribed spacer (ITS), including ITS2 and seven chloroplast regions: *mat*K, *rbc*L, *rpo*C1, SLS, *psb*A–*trn*H, *trn*L–F, and *ycf*1 (Table 1). The aligned sequences of the eight DNA barcoding loci ranged from 221 to 938 bp in length. Specifically, the aligned lengths were 705 bp for ITS, 222 bp for ITS2, 847 bp for *mat*K, 708 bp for *rbc*L, 502 bp for *rpo*C1, 294 bp for SLS, 378 bp for *psb*A–*trn*H, 938 bp for *trn*L–F, and 895 bp for *ycf*1. The average G + C content varied among loci, with ITS showing the highest value (62.19%), followed by ITS2 (59.76%), *rbc*L (42.37%), *rpo*C1 (41.63%), *trn*L–F (35.18%), SLS (33.61%), *mat*K (32.94%), *ycf*1 (29.83%), and *psb*A–*trn*H (28.74%). Sequence conservation analysis revealed high proportions of conserved sites across all loci, ranging from 97.16% (ITS) to 100% (*rbc*L and *rpo*C1). Variable sites were detected mainly in ITS (2.84%), ITS2 (2.25%), SLS (2.04%), *ycf*1 (1.12%), *psb*A–*trn*H (1.06%), *mat*K (0.47%), and *trn*L–F (0.32%), whereas no variable sites were observed in *rbc*L and *rpo*C1. Parsimony-informative sites were present in ITS (1.42%), SLS (1.36%), ITS2 (0.90%), *psb*A–*trn*H (0.79%), *ycf*1 (0.67%), *trn*L–F (0.32%), and *mat*K (0.35%), while none were detected in *rbc*L and *rpo*C1. In contrast, singleton sites were observed primarily in ITS (1.42%), ITS2 (1.35%), SLS (0.68%), *ycf*1 (0.45%), and *psb*A–*trn*H (0.26%). Notably, no intraspecific variation was detected across all loci, whereas interspecific variation was highest in ITS (1.29%), followed by ITS2 (0.96%), SLS (0.85%), *ycf*1 (0.52%), *psb*A–*trn*H (0.57%), *mat*K (0.24%), and *trn*L–F (0.17%), with *rbc*L and *rpo*C1 showing no interspecific variation.

Based on the interspecific variation analysis, the ITS region exhibited the highest level of variation among all DNA barcoding loci examined. When the nucleotide sequences of *Mitragyna speciosa* were compared with those of allied *Mitragyna* species, namely *M. diversifolia*, *M. hirsuta*, and *M. rotundifolia*, clear sequence divergence was observed in the ITS region. In the ITS alignment (705 bp), no intraspecific variation was detected among the three *M. speciosa* variants (Kan Daeng, Kan Khiao, and Hang Kang), which shared an identical ITS haplotype. The allied *Mitragyna* species exhibited clear ITS divergence from *M. speciosa*, *M. diversifolia* and *M. hirsuta* shared the same ITS haplotype and differed from *M. speciosa* by 13 diagnostic sites (10 SNPs and 3 indels), whereas *M. rotundifolia* showed 19 diagnostic differences (16 SNPs and 3 indels). Key discriminatory positions included SNPs at sites 89–90, 138–140, 202, and 612, together with informative indels at positions 144, 482, and 536. These fixed and species-specific characters support the utility of ITS as a robust barcode locus for authenticating *M. speciosa* and distinguishing it from closely allied *Mitragyna* species.

**Figure 2:**
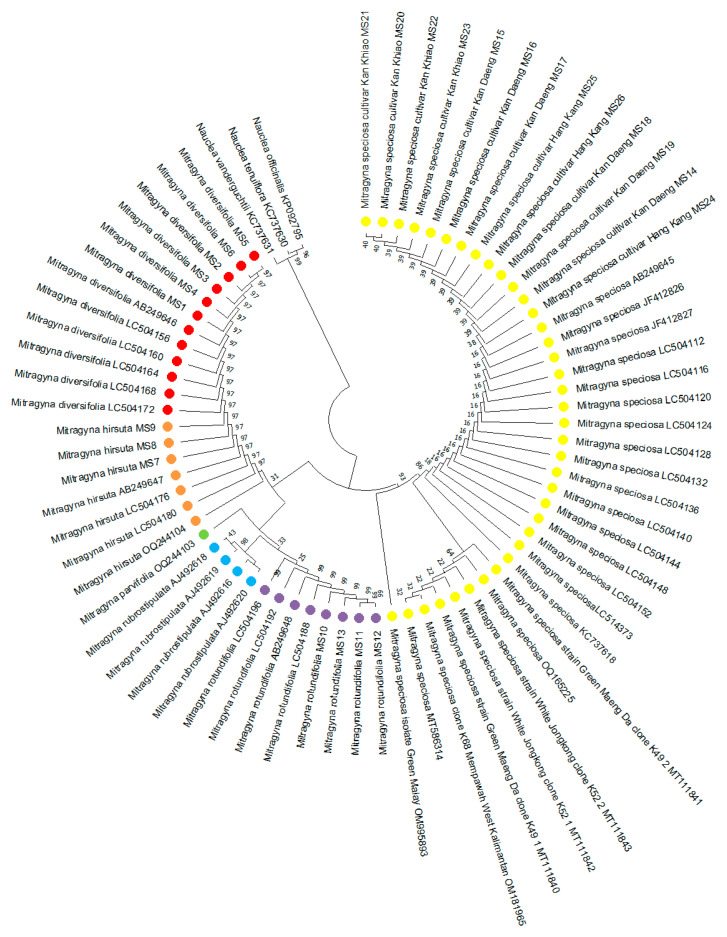
Neighbor-Joining phylogenetic tree of *Mitragyna* species based on ITS sequences. The Neighbor-Joining (NJ) phylogenetic tree was constructed based on internal transcribed spacer (ITS) nucleotide sequences to infer the phylogenetic relationships among *Mitragyna* species. The analysis included three *Mitragyna speciosa* variants (highlighted in yellow) (Kan Daeng, Kan Khiao, and Hang Kang) together with allied species, namely *M. diversifolia* (red), *M. hirsuta* (orange), and *M. rotundifolia* (purple). Additional ITS sequences of *Mitragyna* retrieved from the GenBank database (*M. parvifolia* (green) and *M. rubrostipulata* (blue)) were incorporated to improve phylogenetic resolution. Species of the genus Nauclea (*N. officinalis*, *N. tenuiflora*, and *N. vanderguchtii*) were used as outgroups to root the tree.

Phylogenetic relationships among species within the genus *Mitragyna* were investigated using internal transcribed spacer (ITS) sequences and reconstructed using the Neighbor-Joining (NJ) method (Figure 2). The dataset comprised ITS sequences newly generated in this study from three *M. speciosa* variants, namely Kan Daeng, Kan Khiao, and Hang Kang, together with closely allied *Mitragyna* species (*M. diversifolia*, *M. hirsuta*, and *M. rotundifolia*). To enhance phylogenetic resolution and place the analyzed taxa within a broader evolutionary context, additional ITS sequences of *Mitragyna* species were retrieved from the GenBank database. Species of the genus *Nauclea* (*N. officinalis*, *N. tenuiflora*, and *N. vanderguchtii*) were selected as outgroups based on their phylogenetic proximity within the Rubiaceae family. The NJ phylogenetic tree resolved the analyzed taxa into distinct and well-supported clades that were consistent with current species delimitations. All *M. speciosa* samples clustered together in a strongly supported monophyletic group, indicating a high degree of genetic similarity among the three variants examined. The absence of internal branching within the *M. speciosa* clade further suggests that no detectable intraspecific divergence was present in the ITS region, supporting the genetic homogeneity of these variants at the nuclear ribosomal level. This result is consistent with the conserved nature of ITS sequences within species while still retaining sufficient variability for interspecific discrimination. In contrast, allied *Mitragyna* species formed separate lineages outside the *M. speciosa* clade. Notably, *M. diversifolia* and *M. hirsuta* were grouped together as a sister clade, reflecting their close genetic relationship and shared ITS sequence characteristics. *M. rotundifolia*, however, was resolved as a distinct lineage, separated from both *M. speciosa* and the clade (*M. diversifolia*–*M. hirsuta*), indicating a greater degree of genetic divergence within the genus. The clear separation among species-level clades highlights the discriminatory power of the ITS region and its suitability for resolving phylogenetic relationships within *Mitragyna*. Overall, the ITS-based NJ phylogeny demonstrated strong effectiveness in distinguishing *M. speciosa* from closely allied *Mitragyna* species and provided a robust molecular framework for species authentication. These findings support the use of ITS sequences as a reliable DNA barcode for phylogenetic inference, taxonomic verification, and quality control of kratom (*M. speciosa*) raw materials and derived products.

### 2.2. Quantification of Mitragynine in Ethanolic Extracts of Kratom Variations and Other Mitragyna Species

**Figure 3:**
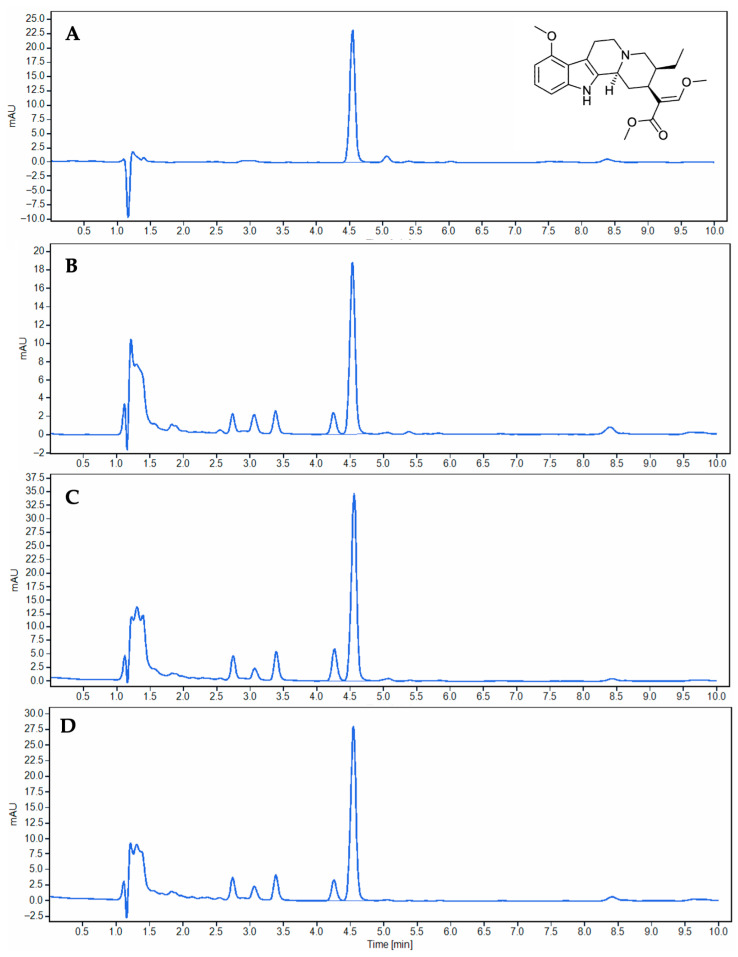
Representative UHPLC chromatograms of mitragynine in kratom crude extracts and mitragynine standard: (**A**) mitragynine standard (60 µg/mL), (**B**) Kan Daeng, (**C**) Kan Khiao, and (**D**) Hang Kang. Mitragynine was consistently detected at a retention time of 4.53–4.58 min.

Although numerous published studies have employed mitragynine as a key chemical marker for the identification of kratom and kratom-derived products [19,20,21,22,23], it is equally important to verify the presence or absence of this compound in other species within the genus *Mitragyna*. Such verification is essential to ensure the specificity and reliability of mitragynine as a diagnostic marker and to avoid potential misidentification arising from closely allied taxa. Ultra-high-performance liquid chromatography (UHPLC) was employed to quantify mitragynine in various kratom variants and allied *Mitragyna* species extracts. Under the optimized chromatographic conditions, mitragynine was consistently eluted at a retention time (RT) of approximately 4.53–4.58 min. Representative chromatograms of crude extracts from the three kratom variants—Kan Daeng, Kan Khiao, and Hang Kang—as well as the mitragynine standard (60 µg/mL), are shown in Figure 3. Each kratom sample exhibited a distinct and well-resolved peak corresponding to mitragynine, whereas this compound was not detected in any of the other *Mitragyna* species analyzed. To further confirm the identity of the detected compound, the UV spectra of the chromatographic peaks in all kratom variants were compared with that of the mitragynine standard. The results demonstrated that the UV absorption profiles of Kan Daeng, Hang Kang, and Kan Khiao were highly consistent with the standard, showing characteristic absorption maxima at similar wavelengths. In addition, the high UV purity values (>99%) observed for all samples support that the detected peak corresponds to a single, well-resolved compound, thereby confirming its identity as mitragynine (Figure S1). The mitragynine content in the crude extracts was quantified and expressed as both % *w*/*w* and mg/g of dried sample. Among the three variants, Kan Khiao demonstrated the highest mitragynine concentration (9.43 ± 0.01% *w*/*w*; 94.33 ± 0.14 mg/g), followed by Hang Kang (7.54 ± 0.00%, 75.39 ± 0.05 mg/g) and Kan Daeng (5.01 ± 0.02%, 50.11 ± 0.18 mg/g). The calibration curve for mitragynine quantification exhibited excellent linearity within the concentration range of 10–150 µg/mL, with a correlation coefficient (R^2^) of 0.9998. The linear regression equation was y = 2.1009x + 1.871 (Figure S2). The method exhibited good accuracy, represented by %recovery in the range of 95.0–105.0%, with the standard deviation NMT 1.0% (intra-day precision) and NMT 2.0% (inter-day precision) (Table S1). To assess the robustness of the method regarding temperature variation, analyses were conducted at 25, 28, and 30 °C. The peak area of mitragynine did not show significant differences among the tested temperatures (Figure S3). The analytical method demonstrated high precision and accuracy, as reflected by the low residuals observed across all calibration levels. The residual standard deviation was 1.58, which was used to estimate the method’s sensitivity. Based on the ICH Q2(R1) guideline, the limit of detection (LOD) and limit of quantification (LOQ) were determined to be 2.98 µg/mL and 7.52 µg/mL, respectively, confirming that the developed UHPLC method was sufficiently sensitive and reliable for the detection and quantification of mitragynine within the tested range.

### 2.3. Characterization of Volatile Phytochemical Constituents in Kratom and Allied Mitragyna Species by Gas Chromatography–Mass Spectrometry (GC-MS)

**Figure 4:**
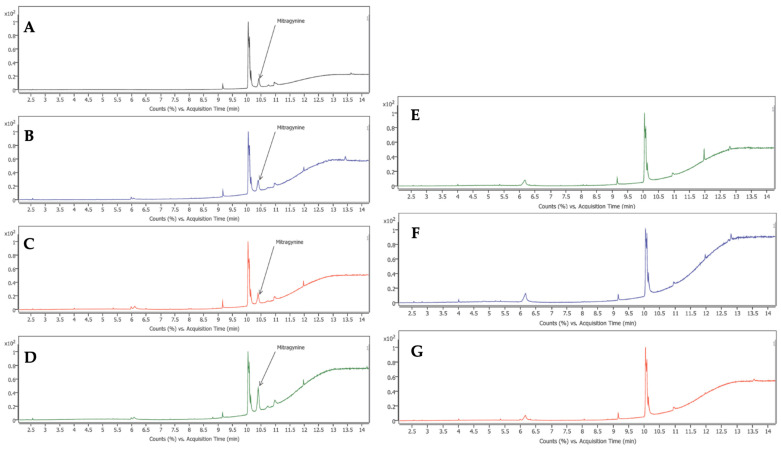
Representative GC-MS Fingerprint of Kratom and *Mitragyna* spp. crude extracts compared with mitragynine standard: (**A**) mitragynine standard, (**B**) Kan Daeng, (**C**) Kan Khiao, and (**D**) Hang Kang, (**E**) *M. diversifolia*, (**F**) *M. hirsuta*, and (**G**) *M. rotundifolia*.

In order to confirm the existence of mitragynine in kratom and the allied *Mitragyna* species, the GC–MS fingerprint chromatograms of different kratom cultivars, namely Kan Daeng, Kan Khiao, and Hang Kang, as well as other *Mitragyna* species, including *M. diversifolia*, *M. hirsuta*, and *M. rotundifolia*, were compared with the mitragynine standard (Figure 4). Under the optimized analytical conditions, the mitragynine reference standard exhibited a distinct peak at a retention time of approximately 10.4 min (Figure 4A). Correspondingly, ethanolic extracts of the kratom cultivars Kan Daeng (Figure 4B), Kan Khiao (Figure 4C), and Hang Kang (Figure 4D) showed a prominent peak at the same retention time (10.4 min), confirming the presence of mitragynine. In contrast, this characteristic mitragynine peak was not detected in the chromatograms of other *Mitragyna* species, including *M. diversifolia* (Figure 4E), *M. hirsuta* (Figure 4F), and *M. rotundifolia* (Figure 4G).

### 2.4. Phytochemical Profile of Kratom Variations and Other Mitragyna Species Using Liquid Chromatography–Mass Spectrometry–Quadrupole Time-of-Flight (LC-MS/QTOF)

The chemical constituents of ethanolic leaf extracts from kratom variants—Kan Daeng, Kan Khiao, and Hang Kang—and allied *Mitragyna* species (*M. diversifolia*, *M. hirsuta*, and *M. rotundifolia*) were comprehensively characterized using LC-MS/QTOF analysis in both positive and negative ionization modes (Tables S2–S4). Phytochemical profiling revealed 94 compounds in positive ionization mode and 42 compounds in negative mode. The kratom variants were characterized by an alkaloid-rich profile, with mitragynine and 7-hydroxymitragynine as the dominant constituents, accompanied by other indole alkaloids such as brucine, corynoxine, gardneramine, hirsutine, and related derivatives. In contrast, *M. diversifolia*, *M. hirsuta*, and *M. rotundifolia* exhibited markedly different chemical profiles, showing negligible or substantially lower levels of mitragynine-type alkaloids. Instead, these non-kratom species were relatively enriched in phenolic acids, flavonoids, and glycosylated metabolites, including chlorogenic acid, caffeoylquinic acid derivatives, rutin, hyperoside, quercetin glycosides, catechin, (–)-epicatechin, procyanidin B1, and triterpenoid-related compounds. Although several polyphenolic metabolites were shared across all *Mitragyna* samples, the pronounced enrichment of mitragynine-type alkaloids in kratom provided a clear chemical basis for discriminating kratom variants from *M. diversifolia*, *M. hirsuta*, and *M. rotundifolia*.

**Figure 5:**
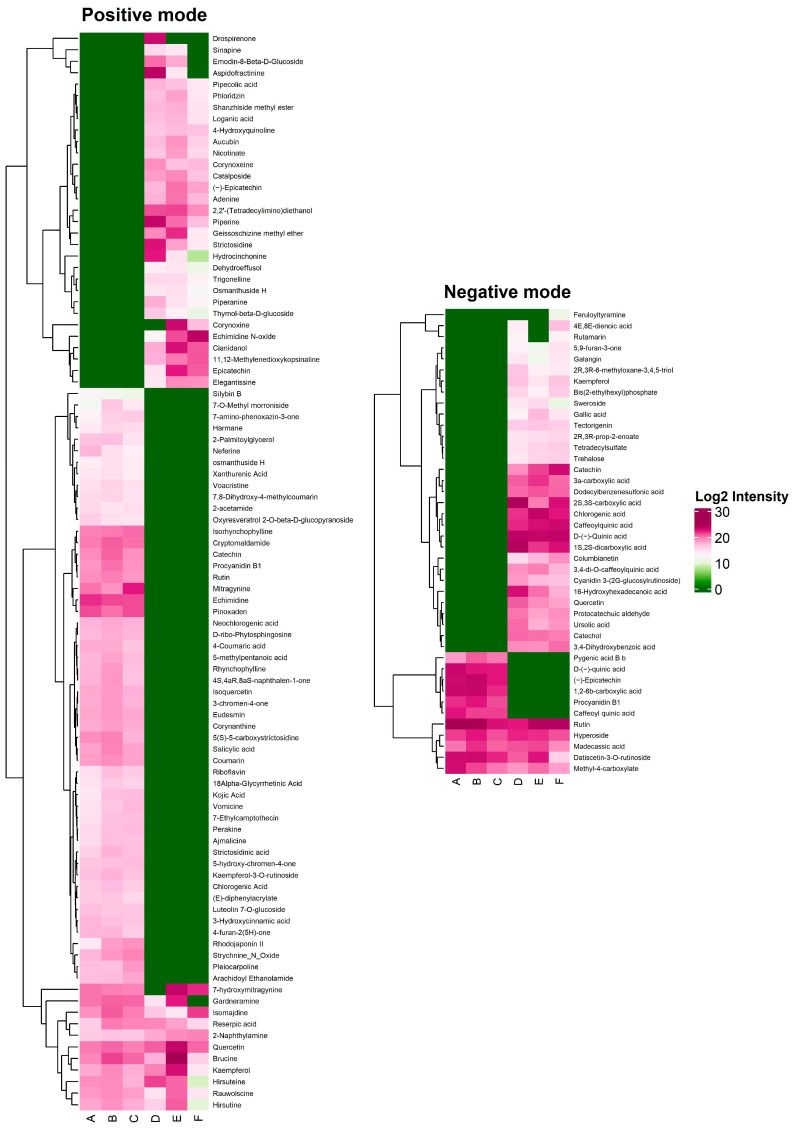
Heat maps based on the percentage area under the curve (%AUC) of chemical constituents obtained from ethanolic leaf extracts of kratom variants—Kan Khiao (**A**), Kan Daeng (**B**), and Hang Kang (**C**)—and allied *Mitragyna* species, including *M. diversifolia* (**D**), *M. hirsuta* (**E**), and *M. rotundifolia* (**F**), as analyzed by LC-MS/QTOF in both positive and negative ionization modes.

**Figure 6:**
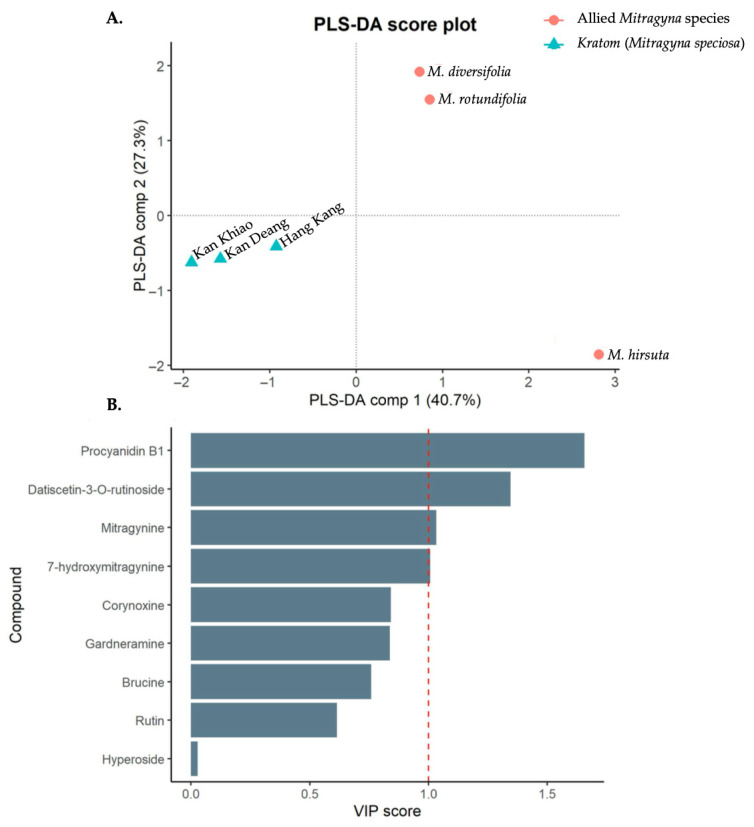
PLS-DA score plot (**A**) illustrating the discrimination between kratom variants and allied *Mitragyna* species based on AUC-derived chemical profiles, and VIP analysis (**B**) identifying key compounds contributing to class separation, with VIP > 1 considered significant.

Heatmaps constructed from the percentage area under the curve (%AUC) values revealed a clear separation between kratom variants and allied *Mitragyna* species in both positive and negative ionization modes (Figure 5). Hierarchical clustering analysis showed that samples corresponding to kratom variants Kan Daeng, Kan Khiao, and Hang Kang formed a distinct cluster driven primarily by high-intensity alkaloid signals, particularly mitragynine and 7-hydroxymitragynine. Conversely, *M. diversifolia*, *M. hirsuta*, and *M. rotundifolia* clustered separately and were characterized by higher relative contributions from phenolic acids, flavonoid glycosides, and non-alkaloid constituents. This contrasting chemotype pattern highlights the robustness of LC-MS/QTOF–based heat map analysis for species-level discrimination within the genus *Mitragyna*. Based on these observations, nine discriminatory compounds—mitragynine, 7-hydroxymitragynine, brucine, corynoxine, gardneramine, rutin, hyperoside, datiscetin-3-O-rutinoside, and procyanidin B1—were subsequently subjected to Partial Least Squares–Discriminant Analysis (PLS-DA) and Variable Importance in Projection (VIP) to further validate the clustering trends and to identify key metabolites responsible for the differentiation between kratom variants and allied *Mitragyna* species. PLS-DA showed a clear discrimination between kratom variants and allied *Mitragyna* species in the score plot (Figure 6A). The samples formed two well-defined clusters corresponding to their respective groups, demonstrating that the AUC-based chemical profiles were sufficient to differentiate between kratom variants and allied *Mitragyna* species. The first two PLS-DA components explained 40.7% and 27.3% of the total variance, respectively, highlighting the strong discriminatory power of the model. To identify the compounds most responsible for the observed group separation, VIP scores were calculated from the PLS-DA model (Figure 6B). The complete profiling data, including FC, Log2FC, and VIP scores, are summarized in Table S6. Among the nine metabolites, five were present at higher levels in the kratom group. Mitragynine was the most prominent kratom-enriched metabolite, exhibiting the highest fold change of 14.85, and was not detected (ND) in the allied *Mitragyna* species. Procyanidin B1 was similarly exclusive to the kratom group and showed the highest VIP score among all metabolites, indicating that it was the strongest contributor to group discrimination in the PLS-DA model. Datiscetin-3-O-rutinoside was approximately 3-fold higher in kratom than in allied *Mitragyna* species. Rutin and hyperoside were also slightly elevated in the kratom variants but showed relatively low VIP scores, suggesting minimal contribution to overall group separation. Conversely, four metabolites were present at substantially higher levels in the allied *Mitragyna* species. Gardneramine and corynoxine were exclusively detected in the allied *Mitragyna* species (ND in kratom), with Log2FC values of −8.03 and −6.62, respectively. 7-Hydroxymitragynine, a pharmacologically important oxidative derivative of mitragynine, was approximately 10-fold higher in allied *Mitragyna* species than in kratom, exceeding the VIP threshold for significance. Brucine was also markedly enriched in allied *Mitragyna* species with roughly an 8.7-fold higher abundance. Notably, four metabolites exceeded the VIP threshold of 1.0, indicating their key roles in discriminating between the two variants: procyanidin B1, datiscetin-3-O-rutinoside, mitragynine, and 7-hydroxymitragynine. These findings highlight distinct alkaloid and flavonoid profiles between kratom and allied *Mitragyna* species, with mitragynine characterizing the kratom variant and 7-hydroxymitragynine, along with several indole and oxindole alkaloids, being more abundant in the allied *Mitragyna* species.

## 3. Discussion

Although the legal status of kratom (*Mitragyna speciosa*) varies across countries, where it is classified as either a controlled substance or a legally permitted product, global demand for kratom has continued to rise rapidly. This increasing popularity is largely driven by expectations of potential health benefits and its psychoactive properties [18]. As observed with many medicinal plants, a surge in market demand is frequently accompanied by an increased risk of adulteration, substitution, or mislabeling of raw materials and commercial products [24,25]. Consequently, these factors have raised significant concerns regarding the purity, authenticity, and quality control of kratom products currently available on the market. DNA barcoding is a rapid and accurate molecular approach for species identification based on standardized DNA regions, including nuclear markers such as the internal transcribed spacer (ITS) and chloroplast markers such as *mat*K, *rbc*L, and *psb*A–*trn*H. This technique plays a critical role in the authentication of herbal materials used in foods and beverages, particularly in detecting adulteration involving closely allied species or species from different plant families [26,27]. Consequently, DNA barcoding has been widely applied to determine the botanical origin of kratom for forensic purposes [28,29,30,31,32,33,34]. In this study, nine target DNA barcoding regions—including the internal transcribed spacer (ITS), maturase K (*mat*K), *psb*A–*trn*H intergenic spacer, ribulose-1,5-bisphosphate carboxylase/oxygenase large subunit (*rbc*L), *rpo*C1, *trn*L–F, *ycf*1, as well as the Secologanin synthase 2 (SLS2) gene—were analyzed to identify the most effective markers for species authentication of kratom and for discriminating kratom from closely allied *Mitragyna* species. The results of this study demonstrated that, based on the percentage of interspecific variation, the internal transcribed spacer (ITS) region exhibited the highest discriminatory power, followed by the ITS2 region. Both markers were found to be suitable for distinguishing kratom from closely allied *Mitragyna* species. These findings are consistent with the results reported by Graham and colleagues [32]. Accordingly, the ITS region has been applied for the authentication of the botanical origin of kratom using various molecular techniques, such as polymerase chain reaction–restriction fragment length polymorphism (PCR–RFLP) [33,34]. In addition, the ITS2 region has been effectively utilized in alternative approaches, including DNA barcoding coupled with high-resolution melting analysis (Bar-HRM) [28]. Overall, DNA barcoding provides a robust, rapid, and reliable molecular tool for authenticating the botanical origin of kratom and discriminating it from closely allied *Mitragyna* species. In particular, the ITS and ITS2 regions demonstrate high discriminatory power, supporting their application in quality control, forensic identification, and regulatory monitoring of kratom materials and products. However, it is important to note that the present study is based on a limited number of samples collected from specific geographic locations in Thailand. Given that intraspecific genetic variation may occur across different ecological regions, broader sampling across diverse geographic origins would further strengthen the reliability and universality of the proposed DNA barcoding markers. Future studies incorporating larger sample sizes and wider geographic coverage are therefore recommended to validate these findings and to better capture potential population-level variation.

To authenticate kratom and distinguish it from other *Mitragyna* species for quality control purposes, chemical profiling has been employed to analyze characteristic chemical markers, particularly in cases where molecular techniques have limitations. Among these, mitragynine is the most commonly used marker for kratom identification and has been widely reported in previous scientific literature [35,36,37]. Several analytical techniques have been employed for the analysis of mitragynine, including high-performance thin-layer chromatography (HPTLC) [36], high-performance liquid chromatography (HPLC) [38], ultra-high-performance liquid chromatography (UHPLC) [38], gas chromatography (GC) [37,39], liquid chromatography (LC) [40,41,42], and surface-enhanced Raman spectroscopy (SERS) [43]. In this study, UHPLC was employed to quantify mitragynine in different kratom cultivars, namely Kan Daeng, Kan Khiao, and Hang Kang, as well as in allied *Mitragyna* species. The results demonstrated that mitragynine was detected exclusively in kratom samples, whereas it was absent in the other *Mitragyna* species examined. Comparative analysis among kratom cultivars revealed that Kan Khiao contained the highest concentration of mitragynine, followed by Hang Kang and Kan Daeng. These findings differ from common claims suggesting that the Kan Daeng cultivar exhibits stronger effects [44,45]. However, Sengnon et al. reported no statistically significant difference in mitragynine content between Kan Khiao and Kan Daeng [22]. This high mitragynine yield in Kan Khiao (9.43 ± 0.01% *w*/*w*) highlights the potential of specific Thai cultivars for pharmaceutical applications and emphasizes that leaf morphology is not a definitive proxy for alkaloid concentration. Such scientific evidence is crucial for correcting traditional misconceptions regarding potency and reinforces the necessity of analytical-based quality control over visual identification. Furthermore, previous studies have indicated that mitragynine levels may vary depending on seasonal factors [22] and geographical origin [20,22]. The present study included a relatively limited number of samples and cultivars, which may not fully represent the chemical diversity of kratom across different growing regions. Environmental factors such as soil composition, climate, season and cultivation practices may significantly influence alkaloid profiles. Therefore, future investigations incorporating larger sample sizes and broader geographic sampling are essential to comprehensively assess chemical variability and to establish more representative quality control standards. Furthermore, to validate the UHPLC chemical profiling results, gas chromatography–mass spectrometry (GC–MS) analysis was performed on kratom cultivars and other *Mitragyna* species and compared with a mitragynine standard. The GC chromatograms revealed a characteristic mitragynine peak at a retention time of 10.5 min in all kratom cultivars, which corresponded precisely with the mitragynine standard, while this peak was absent in the other *Mitragyna* species examined. These findings are consistent with previous reports indicating distinct GC–MS metabolite profiles among the four *Mitragyna* species occurring in Thailand [46].

In this study, the chemical constituents of ethanolic leaf extracts from kratom variants—Kan Daeng, Kan Khiao, and Hang Kang—and allied *Mitragyna* species (*M. diversifolia*, *M. hirsuta*, and *M. rotundifolia*) were comprehensively characterized using LC-MS/QTOF analysis in both positive and negative ionization modes. Kratom variants exhibited an alkaloid-rich profile dominated by mitragynine and 7-hydroxymitragynine, along with other indole alkaloids such as brucine, corynoxine, and gardneramine, whereas *M. diversifolia*, *M. hirsuta*, and *M. rotundifolia* showed markedly different profiles with negligible or substantially lower levels of mitragynine-type alkaloids. Consistent with previous literature, LC–MS/QTOF analysis in positive ionization mode has been reported to detect mitragynine as one of the major constituents, further supporting the reliability of this analytical approach for kratom characterization [47]. Rachid et al. analyzed kratom alkaloids in the foliage and flowers of kratom using LC–MS/MS and reported the presence of several major alkaloids, including mitragynine, speciogynine, speciociliatine, mitraciliatine, corynantheidine, paynantheine, 7-hydroxymitragynine, ajmalicine, and mitraphylline [42]. In the present study, although LC–MS/QTOF was employed under different analytical conditions, comparable kratom alkaloids—such as mitragynine, 7-hydroxymitragynine, corynantheidine, and ajmalicine—were consistently detected, supporting the robustness of high-resolution mass spectrometry for kratom alkaloid profiling. Therefore, LC–MS/MS and LC–MS/QTOF have been widely applied for the analysis of products suspected of containing kratom or kratom alkaloids [40,48,49], as well as for the detection of specific kratom alkaloids—particularly mitragynine and 7-hydroxymitragynine—in biological matrices such as hair [50], urine [51,52], plasma [41], and umbilical cord [53] samples. Collectively, these results demonstrate that LC–MS/QTOF is a powerful analytical approach for differentiating kratom variants from closely allied *Mitragyna* species and highlight substantial interspecific chemical divergence within the genus *Mitragyna*. Heat map visualization and hierarchical clustering based on %AUC values clearly separated kratom variants (Kan Daeng, Kan Khiao, and Hang Kang) from allied *Mitragyna* species, with the former clustering driven primarily by high-intensity alkaloid signals and the latter characterized by higher contributions from phenolic acids and flavonoid glycosides. Multivariate analysis using partial least squares–discriminant analysis (PLS–DA) of nine selected discriminatory compounds further confirmed the clear separation between kratom variants and allied *Mitragyna species*. Variable importance in projection (VIP) analysis identified procyanidin B1 and datiscetin-3-O-rutinoside as the most influential markers, followed by mitragynine and 7-hydroxymitragynine, highlighting their key roles in species differentiation. The identification of these compounds as high-scoring VIP markers emphasizes their significance in forensic diagnostics. Since procyanidin B1 and datiscetin-3-O-rutinoside are highly enriched in allied species but negligible in *M. speciosa*, they serve as effective negative markers; their detection in commercial products labeled as kratom would provide definitive evidence of adulteration or substitution with non-mitragynine-producing *Mitragyna* species. Notably, procyanidin B-type compounds have previously been reported in kratom [47,54,55], and earlier studies have demonstrated that procyanidin B exhibits virucidal activity against SARS-CoV-2 [54], suggesting potential additional biological relevance of this compound beyond its chemotaxonomic value. Therefore, the present study demonstrates that procyanidin B1, datiscetin-3-O-rutinoside, mitragynine, and 7-hydroxymitragynine represent promising chemical markers for discriminating kratom from other *Mitragyna* species occurring in Thailand, supporting their application in the quality control of kratom materials as well as in forensic identification.

A single conventional analytical approach for the identification or authentication of herbal materials, particularly in quality control and forensic applications, may be prone to inaccuracies due to various factors that can lead to ambiguous or inconclusive results. Therefore, the integration of multiple analytical methods is recommended to enhance the reliability and validity of the findings. Several studies have demonstrated the effectiveness of combining DNA barcoding with chemical profiling techniques such as LC–MS, which provides a more robust and accurate approach for authentication. This integrative strategy has been successfully applied in the authentication of true nutmeg (*Myristica fragrans*) [56], medicinal materials from *Uncaria* species [57], commercial Radix Astragali (*Astragalus* spp.) [58], and *Salix alba* L. stem bark [59]. Accordingly, in the present study, the applicability of the proposed workflow for routine laboratory use in kratom materials and related products is further supported by its integrated molecular–chemical approach, which enhances both specificity and analytical confidence. Conventional identification of kratom primarily relies on targeted chemical analysis, particularly the detection of mitragynine using GC–MS or LC–MS techniques [40,48,49]. While these methods are highly sensitive, they may be limited in cases involving highly processed or degraded samples, where alkaloid content is reduced or altered, potentially leading to inconclusive results. In contrast, DNA barcoding enables species-level identification even in trace or degraded plant materials [26,27], providing an additional and independent line of evidence. Moreover, the incorporation of metabolomic profiling combined with multivariate analysis enhances discriminatory power by integrating both positive markers (e.g., mitragynine and 7-hydroxymitragynine) and negative markers (e.g., procyanidin B1 and datiscetin-3-O-rutinoside). Compared to single-method approaches currently used in routine practice, this integrated workflow improves analytical reliability, specificity, and the detection of adulteration involving closely allied species. Therefore, the proposed approach is well-suited for routine applications, particularly in regulatory monitoring, product authentication, and forensic investigations of kratom-containing materials.

## 4. Materials and Methods

### 4.1. Plant Material Collection and Morphological Identification

Leaf samples of kratom (*Mitragyna speciosa*) variants—Kan Daeng, Kan Khiao, and Hang Kang—as well as other *Mitragyna* species native to Thailand, including *M. diversifolia*, *M. hirsuta*, and *M. rotundifolia*, were collected for this study. The plant materials were taxonomically authenticated based on key morphological traits such as leaf, flower, and fruit characteristics by Chatchai Ngernsaengsaruay, Associate Professor of Botany, Kasetsart University, Bangkok, Thailand and Wannaree Charoensup, Botanist of the Faculty of Pharmacy, Chiang Mai University, Chiang Mai, Thailand. The identification was further verified through comparison with the taxonomic descriptions reported by Ngernsaengsaruay et al. [60]. For molecular analysis, fresh leaves of the *M. speciosa* variants—Kan Daeng (MS14-19), Kan Khiao (MS20-23), and Hang Kang (MS24-26)—were collected from established kratom plantations (Table 2). Fresh samples of the other *Mitragyna* species—*M. diversifolia* (MS1-4), *M. hirsuta* (MS7-8), and *M. rotundifolia* (MS10-12)—were obtained from various natural habitats across Thailand, while authenticated dried specimens (MS5-6 for *M. diversifolia*, MS9 for *M. hirsuta*, and MS13 for *M. rotundifolia*) were kindly provided by the Herbarium of the Queen Sirikit Botanic Garden (QBG), Chiang Mai, Thailand. For chemical analysis, *M. speciosa* samples representing the Kan Daeng (MS14) and Kan Khiao (MS20) variants were collected from reputable plantations in Surat Thani Province, whereas the Hang Kang (MS25) variant was obtained from Pathum Thani Province. Leaf samples of *M. diversifolia* (MS1) and *M. hirsuta* (MS7) were collected from Prawet, Bangkok, and *M. rotundifolia* (MS10) was obtained from Chiang Mai University, Chiang Mai, Thailand. All collected samples were air-dried under shade, ground into fine powder, and stored in airtight containers under desiccated conditions until further molecular and chemical analyses were performed.

### 4.2. DNA Barcoding and Sequencing Analysis

Leaf samples of *Mitragyna speciosa* representing three morphological variations, along with *M. diversifolia*, *M. hirsuta*, and *M. rotundifolia*, were ground into fine powder using a mortar and pestle with the aid of liquid nitrogen to preserve DNA integrity. Genomic DNA was extracted from approximately 100 mg of powdered tissue using the DNeasy Plant Mini Kit (Qiagen, Hilden, Germany) according to the manufacturer’s instructions. Polymerase chain reaction (PCR) amplification was performed on a T960 PCR thermocycler (Drawell, Chongqing, China) to amplify eight target regions used for DNA barcoding: the internal transcribed spacer (ITS), maturase K (*mat*K), *trn*H-*psb*A intergenic spacer, ribulose-1,5-bisphosphate carboxylase/oxygenase large subunit (*rbc*L), *rpo*C1, *trn*L-F, and *ycf*1 regions, as well as the Secologanin synthase 2 (SLS2) genes [61]. Each PCR contained 100–200 ng of genomic DNA, 2× PCR Buffer for KOD FX Neo, nuclease-free water, KOD FX Neo DNA polymerase (TOYOBO Life Science, Osaka, Japan), and forward and reverse primers specific to each marker (primer sequences are listed in Table S6). The thermal cycling parameters for each region are provided in Table S6. Amplification products were confirmed by electrophoresis on 1.8% (*w*/*v*) agarose gels, stained with RedSafe™ nucleic acid staining solution (iNtRON Biotechnology, Seongnam, Republic of Korea), and visualized under UV illumination using a Gel Documentation EZ Imager (Bio-Rad, Hercules, CA, USA). The verified PCR products were subsequently purified and sequenced in both directions using an ABI PRISM 3730 XL Genetic Analyzer (Applied Biosystems, Waltham, MA, USA). The resulting nucleotide sequences were aligned with corresponding reference sequences retrieved from the DDBJ/EMBL/GenBank database using ClustalX software version 2.1. Sequence variation and interspecific divergence among the six universal barcode regions were evaluated in MEGA version 11, employing the Kimura 2-parameter (K2P) model to estimate genetic distances and assess discriminatory power.

### 4.3. Extraction of Plant Materials for Phytochemical Analysis

The extraction procedure for phytochemical analysis was conducted following the method described in our previous study, with minor modifications [19]. In order to preserve heat-sensitive phytoconstituents in *Mitragyna speciosa* leaves, drying was performed at a controlled temperature of 50 °C. The dried leaves were then finely ground using an electric grinder to obtain a uniform powder. Approximately 400 g of the powdered material was extracted with 4000 mL of ethanol (solvent-to-sample ratio of 10:1, *v*/*w*) using a Soxhlet extractor (Haberg, Germany) for 20 h. The extract was subsequently concentrated under reduced pressure using a rotary evaporator to remove the solvent. The resulting crude ethanolic extract was collected in amber glass bottles and stored under refrigerated conditions until further phytochemical analysis.

### 4.4. Determination of Mitragynine Content in Kratom Variation and Its Allied Mitragyna Species Using Ultra-High-Performance Liquid Chromatography (UHPLC)

The Ultra-High-Performance Liquid Chromatography (UHPLC) procedure for mitragynine determination was adapted from previously published protocols [19]. Analyses were conducted on an Agilent 1290 Infinity II Series system (Waldbronn, Germany), equipped with a quaternary pump, autosampler, column thermostat, and photodiode array (PDA) detector. Data acquisition and processing were performed using Agilent OpenLAB CDS software version 2.6 (Agilent, Santa Clara, CA, USA). Chromatographic separation was achieved on a ZORBAX Eclipse Plus C18 Rapid Resolution column (3.0 × 100 mm, 1.8 µm; San Diego, CA, USA). The mobile phase consisted of an isocratic mixture of 10 mM ammonium formate and acetonitrile (65:35, *v*/*v*), delivered at a constant flow rate of 0.3 mL/min. The column was operated at ambient temperature. Detection was set at 225 nm, the optimal wavelength for mitragynine quantification. An injection volume of 5 µL was used for both standards and sample solutions. Calibration and quantification were carried out using a mitragynine reference standard. The retention time and peak areas of analytes were compared against a standard calibration curve generated from known mitragynine concentrations, enabling precise determination of its content in the test samples.

### 4.5. Volatile Phytochemical Analysis in Kratom Variants and Allied Mitragyna Species by Gas Chromatography–Mass Spectrometry (GC-MS)

Gas chromatography–mass spectrometry (GC–MS) was employed to analyze the volatile phytochemical constituents of kratom variants and allied *Mitragyna* species in order to discriminate kratom from other closely allied taxa. GC–MS analyses were performed using an Agilent 7890A gas chromatograph coupled with a 5975C single quadrupole mass selective detector (Agilent Technologies, Wilmington, DE, USA). Data acquisition and processing were carried out using MSD Enhanced MassHunter software version 12.1. Chromatographic separation was achieved on an Agilent HP-5 MS Ultra Inert capillary column (30 m × 0.250 mm i.d., 0.25 µm film thickness). Helium was used as the carrier gas under constant pressure mode at 18.7 psi. The injector was operated in split mode with a split ratio of 20:1, an inlet temperature of 280 °C, and an injection volume of 1 µL. The oven temperature program was set as follows: an initial temperature of 100 °C held for 0.5 min, followed by a ramp of 20 °C min^−1^ to a final temperature of 325 °C, which was held for 2.5 min, resulting in a total run time of 14.25 min. The mass spectrometer was operated in electron ionization (EI) mode with autotune applied to maximize sensitivity across the mass range. The MS transfer line temperature was set to 280 °C, the ion source temperature to 230 °C, and the quadrupole temperature to 150 °C. Full-scan mass spectra were acquired over an *m*/*z* range of 35–400, with a filament delay of 2 min. Mitragynine was analyzed using full-scan mode on the single quadrupole MS. Compound identification was achieved by integrating chromatographic peaks using MassHunter software and comparing the obtained mass spectra with those of the mitragynine reference standard and entries in the NIST mass spectral library. The identity of mitragynine was confirmed based on matching retention time and characteristic fragmentation patterns, ensuring reliable discrimination of kratom from other *Mitragyna* species.

### 4.6. Phytochemical Profiling Analysis of Kratom Variation and Allied Mitragyna Species Using Liquid Chromatography–Mass Spectrometry–Quadrupole Time-of-Flight (LC-MS/QTOF)

The Liquid Chromatography–Mass Spectrometry–Quadrupole Time-of-Flight (LC–MS/QTOF) method employed in this study was adapted from previously reported protocols [62]. Each sample (approximately 10 mg) was accurately weighed and dissolved in 95% ethanol containing 50 ng/mL sulfadimethoxine as the internal standard. The mixture was sonicated for 10 min to ensure complete dissolution, followed by centrifugation at 14,000 rpm for 10 min. The resulting supernatant was carefully transferred into an LC–MS vial and subjected to LC–MS/QTOF analysis. LC–MS analysis was performed using an Agilent 6545XT AdvanceBio UHPLC Quadrupole Time-of-Flight (QTOF) Mass Spectrometer (Agilent Technologies, Santa Clara, CA, USA) equipped with a 1290 Infinity II UHPLC system. The chromatographic separation was achieved on an Agilent Poroshell 120 EC-C18 column (2.1 × 100 mm, 2.7 µm) maintained at 50 °C. The mobile phase consisted of solvent A (0.1% formic acid in water) and solvent B (0.1% formic acid in acetonitrile), delivered at a flow rate of 0.4 mL/min. The injection volume was 10 µL. A linear gradient elution program was applied as follows: 0.0–0.5 min, 100% A; 0.5–10.5 min, 45% A and 55% B; 10.5–12.5 min, 25% A and 75% B; 12.5–14.0 min, 0% A and 100% B; 14.0–17.0 min, 0% A and 100% B; 17.0–17.5 min, 100% A and 0% B; 17.5–20.0 min, 100% A and 0% B. Mass spectrometric detection was conducted using the Agilent 6545XT LC–QTOF operating in high-resolution positive and negative electrospray ionization (ESI) modes. The drying gas temperature was set at 325 °C with a flow rate of 13 L/min, and the sheath gas temperature was 275 °C with a flow rate of 12 L/min. The nebulizer pressure was maintained at 45 psi. The capillary voltage was set to +4000 V in positive mode and −3000 V in negative mode. The isotope width was 1.3 *m*/*z*. For MS acquisition, the mass range was *m*/*z* 40–1700 for MS^1^ and *m*/*z* 25–1000 for MS^2^. Collision energies were set at 20 eV (positive mode) and 10 eV (negative mode). The acquisition rate was 3.35 spectra/s, with a maximum of 10 precursors per cycle and a precursor threshold of 5000 counts. The retention time threshold was 0.001%. Reference ions were continuously introduced for real-time mass calibration: *m*/*z* 121.0509 and 922.0098 (positive mode), and *m*/*z* 112.9856 and 1033.9881 (negative mode). Compound identification was carried out based on accurate mass measurement and MS/MS fragmentation patterns, with comparison against public databases and previously reported literature. Due to the lack of authentic reference standards, the identified compounds were considered as putatively annotated compounds with a high level of confidence based on MS/MS spectral matching.

Putative phytochemical compounds detected by LC–MS/QTOF were further analyzed to discriminate between kratom (*M. speciosa*) and the allied *Mitragyna* samples using hierarchical clustering and supervised multivariate approaches. A hierarchical clustered heatmap was constructed using the ComplexHeatmap package [63] in R to visualize global variations in compound abundance and sample similarity. This analysis enabled unsupervised grouping of samples and highlighted differential metabolite patterns associated with each plant type. To further enhance class discrimination, supervised multivariate analysis was performed using partial least squares–discriminant analysis (PLS-DA) implemented in the mixOmics package [64]. The PLS-DA model was used to maximize separation between kratom and the other *Mitragyna* species based on their phytochemical profiles and to identify compounds responsible for the observed differences. Differential metabolites between kratom and allied *Mitragyna* species were evaluated using fold change (FC), log2 fold change (Log2FC), and variable importance in projection (VIP) scores derived from PLS-DA. Zero values were replaced with half the minimum non-zero detected value prior to FC calculation. VIP scores were used to rank compounds according to their contribution to class separation. Compounds with VIP values greater than 1 were considered significant discriminatory markers. These predominant compounds were interpreted as key phytochemical signatures underlying the differentiation between kratom and non-kratom samples and were regarded as potential chemical markers for authentication and classification.

## 5. Conclusions

**Figure 7:**
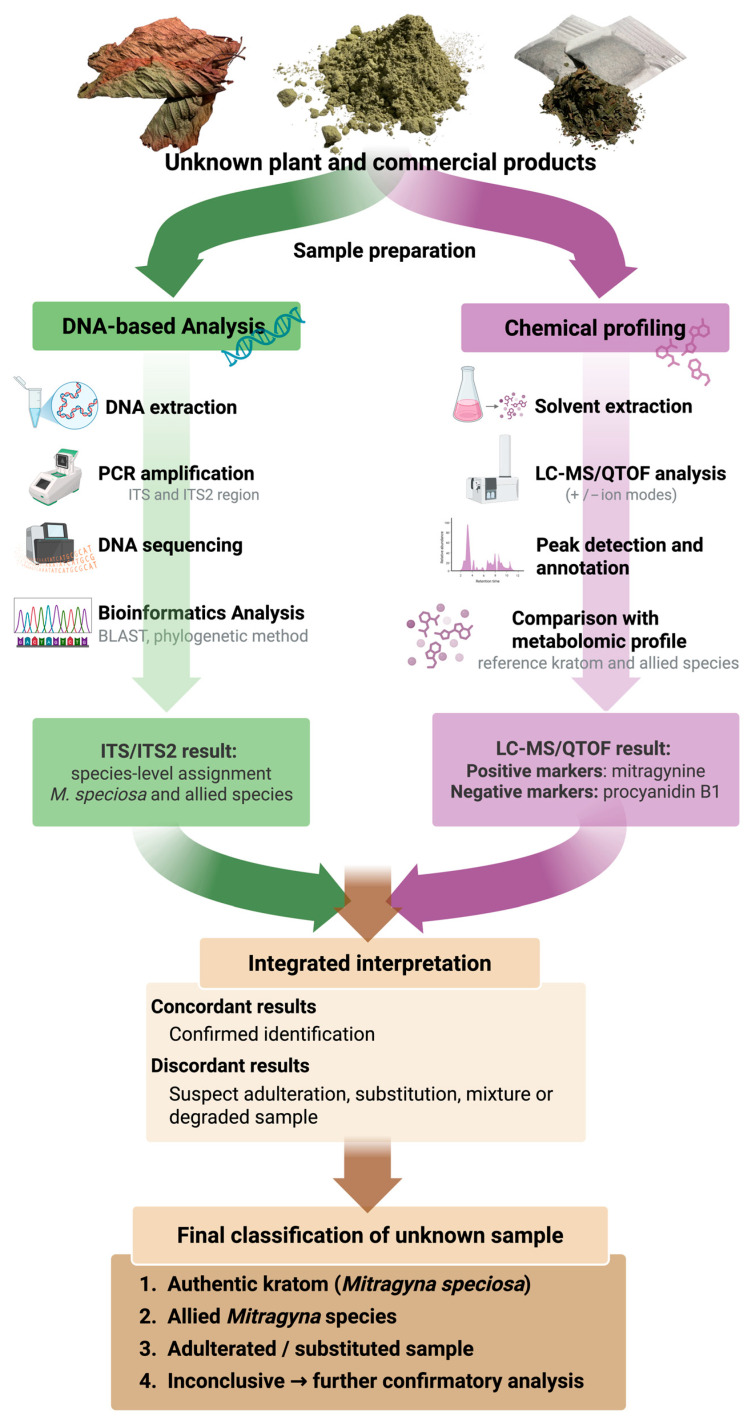
Schematic workflow for the integrated identification of unknown samples using ITS/ITS2 DNA barcoding and LC-MS/QTOF metabolomic profiling. The unknown sample is analyzed using two complementary approaches: ITS/ITS2 DNA barcoding for species-level authentication and LC-MS/QTOF metabolomic profiling for chemical discrimination. Final identification is achieved by integrating molecular evidence with diagnostic chemical markers, allowing confirmation of authentic kratom, discrimination from allied *Mitragyna* species, and detection of adulteration or substitution. Created using BioRender (Intharuksa, A., 2026). Available online: https://BioRender.com/r5mdqc3 (accessed on 31 January 2026).

This study establishes an integrated molecular and chemical approach for the reliable authentication and quality control of kratom (*Mitragyna speciosa*) and its discrimination from closely allied *Mitragyna* species in Thailand. DNA barcoding identified the ITS and ITS2 regions as the most informative markers for species-level identification, while complementary UHPLC, GC–MS, and LC–MS/QTOF analyses confirmed that mitragynine was detected exclusively in kratom variants and absent in allied species. Metabolomic profiling further revealed an alkaloid-rich chemotype in kratom, dominated by mitragynine and 7-hydroxymitragynine, in contrast to the phenolic- and flavonoid-enriched profiles of non-kratom *Mitragyna* species. Multivariate analysis highlighted procyanidin B1, datiscetin-3-O-rutinoside, mitragynine, and 7-hydroxymitragynine as key discriminatory markers supporting chemotaxonomic classification, quality assurance, and forensic identification. The complementary integration of ITS/ITS2 DNA barcoding and LC–MS/QTOF metabolomic profiling enables robust discrimination between authentic kratom, closely allied species, and adulterated samples, thereby enhancing the reliability of quality control for kratom raw materials and supporting forensic applications (Figure 7). Future work should integrate genome-scale barcoding with advanced metabolomics and machine-learning tools and develop rapid, field-deployable assays to strengthen regulatory monitoring and ensure the safety and authenticity of kratom products in global markets for the herbal industrial and forensic applications.

## Figures and Tables

**Table 1:** Sequence data analysis of different DNA barcoding gene loci of kratom (*M. speciosa*) variation and *Mitragyna* spp.

DNA Barcoding Gene Loci	ITS	ITS2	*mat*K	*rbc*L	*rpo*C1	SLS	*psb*A–*trn*H	*trn*L–F	*ycf*1
Length range (bp)	703–704	221–222	847	708	502	293–294	365–378	938	895
Aligned length (bp)	705	222	847	708	502	294	378	938	895
Average G + C (%)	62.19	59.76	32.94	42.37	41.63	33.61	28.74	35.18	29.83
C (%)	685 (97.16)	217 (97.74)	843 (99.53)	708 (100.00)	502 (100.00)	288 (97.96)	374 (98.94)	935 (99.68)	885 (98.88)
V (%)	20 (2.84)	5 (2.25)	4 (0.47)	0 (0.00)	0 (0.00)	6 (2.04)	4 (1.06)	3 (0.32)	10 (1.12)
Pi (%)	10 (1.42)	2 (0.90)	3 (0.35)	0 (0.00)	0 (0.00)	4 (1.36)	3 (0.79)	3 (0.32)	6 (0.67)
S (%)	10 (1.42)	3 (1.35)	1 (0.12)	0 (0.00)	0 (0.00)	2 (0.68)	1 (0.26)	0 (0.00)	4 (0.45)
Interspecific variation (%)	1.29	0.96	0.24	0.00	0.00	0.85	0.57	0.17	0.52

C: conserve sites, V: variable sites, Pi: parsimony-informative sites, S: singleton sites.

**Table 2:** Lists of samples of *Mitragyna* species distributed in Thailand and *M. speciosa* variation.

*Mitragyna* Species		Code	Regions	Locality (District, Province)
*M. diversifolia*		MS1	North	Ban Tak, Tak
		MS2	North-East	Mueang, Maha Sarakham
		MS3	North-East	Kantharawichai, Maha Sarakham
		MS4	North-East	Ban Khwao, Chaiyaphum
		MS5	North-East	Mueang, Udon Thani
		MS6	West	Si Sawat, Kanchanaburi
*M. hirsuta*		MS7	Central	Prawet, Bangkok
		MS8	Central	Prawet, Bangkok
		MS9	North	Gulyani Vadhana, Chiang Mai
*M. rotundifolia*		MS10	North	Mueang Chiang Mai, Chiang Mai
		MS11	North	Mueang Chiang Mai, Chiang Mai
		MS12	North-east	Ban Khwao, Chaiyaphum
		MS13	West	Si Sawat, Kanchanaburi
*M. speciosa*	Kan Daeng	MS14	South	Khiri Rat Nikhom, Surat Thani
		MS15	South	Khiri Rat Nikhom, Surat Thani
		MS16	South	Mueang, Nakhon Si Thammarat
		MS17	South	Mueang, Nakhon Si Thammarat
		MS18	Central	Sam Khok, Pathum Thani
		MS19	Central	Sai Noi, Nonthaburi
	Kan Khiao	MS20	South	Khiri Rat Nikhom, Surat Thani
		MS21	South	Mueang, Nakhon Si Thammarat
		MS22	Central	Sam Khok, Pathum Thani
		MS23	Central	Sai Noi, Nonthaburi
	Hang Kang	MS24	South	Khiri Rat Nikhom, Surat Thani
		MS25	Central	Sam Khok, Pathum Thani
		MS26	Central	Sai Noi, Nonthaburi

## Data Availability

The original contributions presented in this study are included in the article/Supplementary Material. Further inquiries can be directed to the corresponding author.

## Supplementary Materials

The following supporting information can be downloaded at https://www.mdpi.com/article/10.3390/plants15071003/s1, Table S1: Validation parameters of the UHPLC method for mitragynine quantification, including accuracy (% recovery ± SD) and precision (intra-day and inter-day %RSD) at different standard concentrations; Table S2: Phytochemicals identified in kratom extracts of the Kan Daeng, Kan Khiao, and Hang Kang variants using LC–MS/QTOF analysis in positive ion mode; Table S3: Phytochemicals identified in kratom extracts of the Kan Daeng, Kan Khiao, and Hang Kang variants using LC–MS/QTOF analysis in negative ion mode; Table S4: Phytochemicals identified in extracts of allied *Mitragyna* species (*M. diversifolia*, *M. hirsuta*, and *M. rotundifolia*) using LC–MS/QTOF analysis in positive ion mode; Table S5: Phytochemicals identified in extracts of allied *Mitragyna* species (*M. diversifolia*, *M. hirsuta*, and *M. rotundifolia*) using LC–MS/QTOF analysis in negative ion mode; Table S6: Differential metabolites between kratom and allied *Mitragyna* species based on fold change (FC), log_2_ fold change (Log_2_FC), and variable importance in projection (VIP) scores derived from PLS-DA; Table S7: List of DNA barcoding primers used in this study; Figure S1: The identity of mitragynine was confirmed by its UV spectrum and UV purity (%); Figure S2: Calibration curve of standard mitragynine showing linear regression (y = 2.1009x + 1.871) with a coefficient of determination (R2 = 0.9998); Figure S3: Robustness evaluation of the UHPLC method showing the effect of column temperature variation (25, 28, and 30 °C) on the peak area of mitragynine. Refs. [65,66,67,68,69,70,71,72] are cited in the supplementary materials.

## Abbreviations

The following abbreviations are used in this manuscript:

ADR	Adverse Drug Reaction
AUC	Area Under the Curve
Bar-HRM	DNA Barcoding–High-Resolution Melting
bp	Base Pair
COX-2	Cyclooxygenase-2
ESI	Electrospray Ionization
GC	Gas Chromatography
GC–MS	Gas Chromatography–Mass Spectrometry
HPLC	High-Performance Liquid Chromatography
HPTLC	High-Performance Thin-Layer Chromatography
HRM	High-Resolution Melting
ICH	International Council for Harmonisation
ITS	Internal Transcribed Spacer
ITS2	Internal Transcribed Spacer 2
K2P	Kimura 2-Parameter Model
LC	Liquid Chromatography
LC–MS/MS	Liquid Chromatography–Tandem Mass Spectrometry
LC–MS/QTOF	Liquid Chromatography–Quadrupole Time-of-Flight Mass Spectrometry
LOD	Limit of Detection
LOQ	Limit of Quantification
MIA	Monoterpene Indole Alkaloid
NJ	Neighbor-Joining
PCR	Polymerase Chain Reaction
PCR–RFLP	Polymerase Chain Reaction–Restriction Fragment Length Polymorphism
PDA	Photodiode Array Detector
PLS–DA	Partial Least Squares–Discriminant Analysis
QTOF	Quadrupole Time-of-Flight
RT	Retention Time
SERS	Surface-Enhanced Raman Spectroscopy
SLS2	Secologanin Synthase 2
SNP	Single Nucleotide Polymorphism
UHPLC	Ultra-High-Performance Liquid Chromatography
VIP	Variable Importance in Projection
%AUC	Percentage Area Under the Curve
