# Supplementary material for: DNA Barcoding and Chemical Profile Using UHPLC, GC-MS and LC-MS/QTOF of Mitragyna speciosa Variation and Allied Species for Quality Control of Kratom Materials
Source: Plants (Basel). 2026 Mar 25;15(7):1003. doi: 10.3390/plants15071003 (PMC13074985; doi:10.3390/plants15071003)
Supplement: Supplementary file 1 [file plants-15-01003-s001.zip › plants-4187935-supplementary.pdf]

DNA Barcoding and Chemical Profile using UHPLC, GC-MS and LC-MS/QTOF of *Mitragyna speciosa* Variation and Allied Species for Quality Control of Kratom Materials

Phunsuk Anantaworasakul, Warunya Arunotayanun, Siripat Chaichit, Suthiwat Khamnuan, Chatchai Ngernsaengsaruay, Chuda Chittasupho, Nisa Leksungnoen, Mingkwan Na Takuatung, Ruedeemars Yubolphan, Apisada Jiso, Tachpon Techarang and Aekkhaluck Intharuksa

Supplementary materials

Table S1 Validation parameters of the UHPLC method for mitragynine quantification, including accuracy (% recovery ± SD) and precision (intra-day and inter-day %RSD) at different standard concentrations

Standard Concentration (μg/mL)	Accuracy	Precision (%RSD)	
	% Recovery ± SD	Intraday	Inter-day
15	99.94 ± 0.41	0.36	1.74
30	102.41 ± 0.54	0.51	1.02
45	101.74 ± 0.68	0.47	1.37

Table S2 Phytochemicals identified in kratom extracts of the Kan Daeng, Kan Khiao, and Hang Kang variants using LC–MS/QTOF analysis in positive ion mode

Average Rt (min)	Average Mz	Metabolite name	Adduct type	Ontology	Kan Deang	Hang Kang	Kan Keaw
3.085	355.10107	Chlorogenic Acid	[M+H]+	Quinic acids and derivatives	425,602.75	389,880.20	770,497.79
3.093	206.04471	Xanthurenic Acid	[M+H]+	Quinoline carboxylic acids	97,283.60	54,699.39	153,711.15
3.160	433.16833	Osmanthuside H	[M+H]+	O-glycosyl compounds	55,526.15	56,713.88	160,501.78
3.253	143.03365	Kojic Acid	[M+H]+	Pyrones and derivatives	84,366.58	807,248.27	717,693.43
3.899	355.10281	Neochlorogenic acid	[M+H]+	Quinic acids and derivatives	1,157,571.73	1,180,969.12	1,603,693.47
3.911	291.08652	Catechin	[M+H]+	Catechins	4,073,341.46	3,417,957.34	12,295,417.47
3.971	165.05455	3-Hydroxycinnamic acid	[M+H]+	Hydroxycinnamic acids	1,050,163.47	478,750.38	714,245.73
4.087	421.16809	7-O-Methyl morroniside	[M+H]+	O-glycosyl compounds	15,805.95	82,514.68	490,631.34
4.357	139.03903	Salicylic acid	[M+H]+	Salicylic acids	2,075,577.08	1,926,464.87	5,131,163.81
4.428	165.05461	4-Coumaric acid	[M+H]+	Hydroxycinnamic acids	1,485,280.51	531,102.04	1,460,545.52
4.655	483.12677	Silybin B	[M+H]+	Flavonolignans	29,826.88	8,176.94	11,466.11
4.848	193.04961	7,8-Dihydroxy-4-methylcoumarin	[M+H]+	7,8-dihydroxycoumarins	203,531.30	120,558.82	249,333.21
4.896	183.09154	Harmane	[M+H]+	Harmala alkaloids	69,235.99	203,218.68	254,299.22
5.057	595.16577	Kaempferol-3-O-rutinoside	[M+H]+	Flavonoid-3-O-glycosides	635,140.17	593,174.31	1,207,796.01
5.062	287.05502	Kaempferol	[M+H]+	Flavonols	1,636,837.27	1,452,193.43	4,235,515.29

5.069	399.23935	Mitragynine	[M+H]+	Corynanthean-type alkaloids	7,937,672.79	54,397,172.11	2,929,758.04
5.117	449.10776	Luteolin 7-O-glucoside	[M+H]+	Flavonoid-7-O-glycosides	175,212.94	510,904.52	480,615.84
5.168	303.05063	Quercetin	[M+H]+	Flavonols	6,559,793.63	5,879,266.07	11,430,761.38
5.180	611.16229	Rutin	[M+H]+	Flavonoid-3-O-glycosides	3,734,270.05	2,802,465.20	5,833,875.91
5.180	465.10327	Isoquercetin	[M+H]+	Flavonoid-3-O-glycosides	1,586,511.36	1,244,024.67	2,613,282.40
5.252	355.20187	Rauvolscine	[M+H]+	Yohimbine alkaloids	2,655,986.81	2,294,347.23	4,068,967.14
5.263	407.13153	Oxyresveratrol 2-O-beta-D-glucopyranoside	[M+H]+	Stilbene glycosides	350,925.29	109,632.11	145,018.49
5.352	517.21838	Strictosidinic acid	[M+H]+	Terpene glycosides	323,416.43	731,351.53	1,196,714.03
5.371	385.21271	Rhynchophylline	[M+H]+	Indolizidines	1,140,235.94	664,546.86	2,584,635.12
5.615	355.20169	Corynantine	[M+H]+	Yohimbine alkaloids	1,525,423.85	1,487,575.63	2,269,727.00
5.621	413.20752	Gardneramine	[M+H]+	3-alkylindoles	7,888,812.15	11,526,910.30	12,363,853.88
5.850	401.20795	Reserpic acid	[M+H]+	Corynanthean-type alkaloids	359,296.29	4,658,692.00	6,580,479.10
5.996	369.21713	Hirsutine	[M+H]+	Corynanthean-type alkaloids	1,723,944.60	1,407,680.54	3,257,358.15
6.145	429.20313	Isomajdine	[M+H]+	Indolizidines	3,245,096.87	5,890,029.36	14,644,490.08
6.331	385.21329	Isorhynchophylline	[M+H]+	Indolizidines	5,379,893.26	9,714,745.88	6,575,656.47
6.860	351.17026	Perakine	[M+H]+	Ajmaline-sarpagine alkaloids	200,367.25	848,703.13	686,065.24
6.873	400.25876	Cryptolamamide	[M+H]+	Peptides	6,419,386.89	9,099,030.80	13,344,537.31
6.906	398.21851	Echimidine	[M+H]+	Alkaloids and derivatives	37,792,595.16	21,661,950.92	23,020,658.16
7.225	367.20233	Hirsuteine	[M+H]+	Corynanthean-type alkaloids	3,662,036.16	1,181,599.38	3,900,921.72
7.355	413.20789	Pleiocarpine	[M+H]+	Aspidofractine alkaloids	666,175.62	2,518,893.85	641,576.32
7.420	351.17084	Strychnine N-oxide	[M+H]+	Strychnos alkaloids	1,125,742.76	5,257,393.02	2,846,924.81
7.465	401.24164	Pinoxaden	[M+H]+	Phenylpyrazoles	21,484,798.89	21,613,099.99	9,233,625.90
7.663	395.19748	Brucine	[M+H]+	Strychnos alkaloids	4,604,331.49	11,296,913.04	26,002,483.47
7.692	377.15002	7-Ethylcamptothecin	[M+H]+	Camptothecins	141,697.24	688,603.77	630,365.85
7.720	381.18140	Vomicine	[M+H]+	Carbazoles	143,310.84	1,002,357.59	491,579.02

7.775	425.17111	5-hydroxy-7-(2-(4-(2-hydroxyethyl)piperazin-1-yl)-2-oxoethoxy)-2-phenyl-4H-chromen-4-one	[M+H]+	Flavones	542,946.68	792,765.14	752,520.34
7.944	377.14957	Riboflavin	[M+H]+	Flavins	163,221.98	404,305.76	778,992.77
8.052	353.18613	Ajmalicine	[M+H]+	Yohimbine alkaloids	201,370.18	645,018.86	688,100.40
8.309	415.23392	7-hydroxymitragynine	[M+H]+	Corynanthean-type alkaloids	7,770,913.42	4,951,550.33	6,085,554.08
8.672	539.23950	2-((6-((6,7-dimethoxy-3,4-dihydroisoquinolin-2(1H)-yl)methyl)-4-oxo-4H-pyran-3-yl)oxy)-N-(3,4-	[M+H]+	Tetrahydroisoquinoline s	238,517.41	165,437.87	116,339.81
10.125	387.18027	Eudesmin	[M+H]+	Furanoid lignans	1,747,883.88	1,686,835.41	2,597,240.22
10.146	318.30042	D-ribo-Phytosphingosine	[M+H]+	1,3-aminoalcohols	940,651.91	1,105,509.29	1,430,236.48
12.276	625.31940	Neferine	[M+H]+	Benzylisoquinolines	1,109,750.07	44,863.79	159,410.98
12.847	331.28452	2-Palmitoylglycerol	[M+H]+	2-monoacylglycerols	604,254.12	112,424.27	703,347.00
12.865	471.34708	18Alpha-Glycyrrhetinic Acid	[M+H]+	Triterpenoids	131,662.65	294,413.99	415,424.68
12.869	356.35269	Arachidoyl Ethanolamide	[M+H]+	N-acylethanolamines	727,711.42	1,710,398.20	818,318.87
14.760	593.42889	(E)-(3S,10R,13R)-10,13-dimethyl-17-octyl-2,3,4,7,8,9,10,11,12,13,14,15,16,17-tetradecahydro	[M+H]+	Steroid	405,559.22	229,661.74	557,511.54

Table S3 Phytochemicals identified in kratom extracts of the Kan Daeng, Kan Khiao, and Hang Kang variants using LC–MS/QTOF analysis in negative ion mode

Average Rt (min)	Average Mz	Metabolite name	Adduct type	Ontology	Kan Deang	Hang Kang	Kan Keaw
5.143	609.14801	Rutin	[M-H]-	Flavonoid-3-O-glycosides	29,682,752.92	7,691,424.54	25,686,973.01
4.359	289.07294	(-)-Epicatechin	[M-H]-	Catechins	10,385,078.14	5,816,073.08	13,850,023.37
5.429	593.15051	Datiscetin-3-O-rutinoside	[M-H]-	Flavonoid-3-O-glycosides	10,016,920.31	4,641,121.64	10,573,798.99
7.959	793.43872	1,2,6b,9,12a-hexamethyl-4a-[3,4,5-trihydroxy-6-(hydroxymethyl)oxan-2-yl]oxy	[M-H]-	Triterpene saponins	11,265,554.43	4,239,998.85	11,940,468.89
3.904	191.05647	D-(-)-quinic acid	[M-H]-	Quinic acids and derivatives	11,648,455.37	5,663,089.41	6,338,533.67

5.329	463.08759	Hyperoside	[M-H]-	Flavonoid-3-O-glycosides	3,169,472.31	2,283,009.97	5,682,017.43
4.107	577.13458	Procyanidin B1	[M-H]-	Biflavonoids and polyflavonoids	4,100,523.03	2,417,709.00	6,236,507.33
3.904	353.08743	Caffeoyl quinic acid (isomer of 831, 833, 834)	[M-H]-	Quinic acids and derivatives	5,954,189.23	3,059,903.75	3,055,452.01
4.850	403.12418	Methyl (1S,4aR,7aR)-4a-hydroxy-7-(hydroxymethyl)-1-[(2S,3R,4S,5S,6R)-3,4,5-tri	[M-H]-	Iridoid O-glycosides	9,368,757.93	916,124.35	2,845,916.81
11.470	487.34241	Pygenic acid B b	[M-H]-	Triterpenoids	383,853.79	975,306.14	1,807,219.05
9.724	503.33725	Madecassic acid	[M-H]-	Triterpenoids	996,683.23	1,557,760.70	4,034,394.14

Table S4 Phytochemicals identified in extracts of allied *Mitragyna* species (*M. diversifolia*, *M. hirsuta*, and *M. rotundifolia*) using LC–MS/QTOF analysis in positive ion mode.

Average Rt (min)	Average Mz	Metabolite name	Adduct type	Ontology	Krathum_1	Krathum_2	Krathum_3
0.722	341.10831	Trehalose	[M-H]-	O-glycosyl compounds	51,736.31	69,468.18	22,686.95
0.952	191.05524	D-(-)-Quinic acid	[M-H]-	Quinic acids and derivatives	12,559,546.90	13,291,093.99	15,500,996.86
1.956	169.01352	Gallic acid	[M-H]-	Gallic acids	162,574.32	21,432.49	8,838.19
2.896	153.01871	3,4-Dihydroxybenzoic acid	[M-H]-	Hydroxybenzoic acid derivatives	525,075.80	1,248,322.95	569,017.79
2.917	109.02901	Catechol	[M-H]-	Catechols	1,196,611.41	850,187.00	1,340,265.27
3.389	353.08612	Chlorogenic acid	[M-H]-	Quinic acids and derivatives	14,324,036.22	5,879,169.51	3,872,185.83
3.516	137.02364	Protocatechuic aldehyde	[M-H]-	Hydroxybenzaldehydes	313,832.45	532,158.49	1,006,302.20
3.890	353.08649	Caffeoylquinic acid	[M-H]-	Quinic acids and derivatives	8,347,391.96	10,546,652.93	4,540,890.39
4.342	245.08177	Columbianetin	[M-H]-	Angular furanocoumarins	164,409.70	501,929.40	41,164.85
4.345	289.07050	Catechin	[M-H]-	Catechins	2,967,468.30	9,729,724.72	506,211.22
4.761	755.20209	Cyanidin 3-(2G-glucosylrutinoside)	[M-2H]-	Anthocyanidin-3-O-glycosides	149,875.89	111,082.76	410,511.32
4.774	609.14484	Rutin	[M-H]-	Flavonoid-3-O-glycosides	19,562,386.73	20,491,546.20	5,647,990.34

4.832	403.12411	Methyl (1S,4aR,7aR)-4a-hydroxy-7-(hydroxymethyl)-1-[(2S,3R,4S,5S,6R)-…]	[M-H]-	Iridoid O-glycosides	1,221,971.60	341,070.08	441,144.13
5.320	463.08640	Hyperoside	[M-H]-	Flavonoid-3-O-glycosides	4,079,385.39	2,252,446.51	4,689,667.40
5.424	593.14923	Datiscetin-3-O-rutinoside	[M-H]-	Flavonoid-3-O-glycosides	6,521,248.56	63,524.28	1,780,425.40
5.619	357.11853	Sweroside	[M-H]-	O-glycosyl compounds	42,129.06	2,149.74	22,213.32
5.727	623.19812	[(2R,3R,4S,5R,6R)-6-[2-(3,4-dihydroxyphenyl)ethoxy]-3,5-dihydroxy-4-…]	[M-H]-	Coumaric acids and derivatives	35,673.66	48,072.48	26,435.90
5.936	515.11786	3,4-di-O-caffeoylquinic acid	[M-H]-	Quinic acids and derivatives	793,622.84	179,403.32	481,613.95
6.135	427.16052	(2R,3R,4R,5R,6S)-2-[[…]]	[M-H]-	Phenolic glycosides	9,813.34	17,324.43	98,100.00
6.733	312.12296	Feruloyltyramine	[M-H]-	Hydroxycinnamic acids and derivatives	0.00	2,826.19	0.00
6.942	301.03448	Quercetin	[M-H]-	Flavonols	566,574.15	310,455.20	1,789,256.01
6.944	285.04053	Kaempferol	[M-H]-	Flavonols	26,491.55	101,867.97	108,190.73
7.434	955.48907	3a-[(2S,3R,4S,5R,6R)-3,5-dihydroxy-6-[[…]]	[M-H]-	Triterpene saponins	3,825,344.11	1,316,469.75	1,702,889.89
7.640	281.13870	5,9-dihydroxy-7-(hydroxymethyl)-5,7-dimethyl-4,5a,6,8a,9-hexahydro-1H-azuleno[5,6-c]furan	[M-H]-	Terpene lactones	3,358.10	27,375.99	9,165.91
7.668	269.04425	Galangin	[M-H]-	Flavonols	3,167.63	22,418.02	22,838.91
8.408	809.43079	(2S,3S,4S,5R,6R)-6-[[…]]	[M-H]-	Triterpene saponins	866,321.61	7,884,989.98	23,518,531.85
8.870	299.05576	Tectorigenin	[M-H]-	Isoflavones	99,041.20	67,142.14	70,846.91
9.180	793.43640	(1S,2R,4aS,6aR,6bR,10S,12aR)-10-[(2R,3R,4S,5R,6S)-3,4-dihydroxy-6-methyl-5-[(2S,3R,4S,5S,6R)-…]	[M-H]-	Triterpene saponins	3,675,866.12	7,601,674.72	21,042,394.74
9.711	503.33636	Madecassic acid	[M-H]-	Triterpenoids	2,695,211.91	559,985.23	2,192,952.63
12.158	293.17834	Tetradecylsulfate	[M-H]-	Sulfuric acid monoesters	54,837.07	76,477.42	31,415.18
12.771	325.18341	Dodecylbenzenesulfonic acid	[M-H]-	Benzenesulfonic acids and derivatives	2,036,675.56	1,460,017.07	1,381,951.40
13.257	321.22012	Bis(2-ethylhexyl)phosphate	[M-H]-	Dialkyl phosphates	11,507.73	70,579.26	51,729.77
13.695	355.15472	Rutamarin	[M-H]-	Psoralens	0.00	8,440.44	7,720.44
14.119	387.18027	(4E,8E)-10-(4-hydroxy-6-methoxy-7-methyl-3-oxo-1H-	[M-H]-	Terpene lactones	0.00	126,966.80	14,933.93

		2-benzofuran-5-yl)-4,8-dimethyldeca-…					
14.241	271.22754	16-Hydroxyhexadecanoic acid	[M-H]-	Long-chain fatty acids	1,145,927.49	217,472.46	8,096,467.66
14.371	455.35202	Ursolic acid	[M-H]-	Triterpenoids	222,438.89	442,698.93	1,273,028.50

Table S5 Phytochemicals identified in extracts of allied *Mitragyna* species (*M. diversifolia*, *M. hirsuta*, and *M. rotundifolia*) using LC–MS/QTOF analysis in negative ion mode.

Average Rt (min)	Average Mz	Metabolite name	Adduct type	Ontology	Krathum_1	Krathum_2	Krathum_3
0.722	341.10831	Trehalose	[M-H]-	O-glycosyl compounds	51,736.31	69,468.18	22,686.95
0.952	191.05524	D-(-)-Quinic acid	[M-H]-	Quinic acids and derivatives	12,559,546.90	13,291,093.99	15,500,996.86
1.956	169.01352	Gallic acid	[M-H]-	Gallic acids	162,574.32	21,432.49	8,838.19
2.896	153.01871	3,4-Dihydroxybenzoic acid	[M-H]-	Hydroxybenzoic acid derivatives	525,075.80	1,248,322.95	569,017.79
2.917	109.02901	Catechol	[M-H]-	Catechols	1,196,611.41	850,187.00	1,340,265.27
3.389	353.08612	Chlorogenic acid	[M-H]-	Quinic acids and derivatives	14,324,036.22	5,879,169.51	3,872,185.83
3.516	137.02364	Protocatechuic aldehyde	[M-H]-	Hydroxybenzaldehydes	313,832.45	532,158.49	1,006,302.20
3.890	353.08649	Caffeoylquinic acid	[M-H]-	Quinic acids and derivatives	8,347,391.96	10,546,652.93	4,540,890.39
4.342	245.08177	Columbianetin	[M-H]-	Angular furanocoumarins	164,409.70	501,929.40	41,164.85
4.345	289.07050	Catechin	[M-H]-	Catechins	2,967,468.30	9,729,724.72	506,211.22
4.761	755.20209	Cyanidin 3-(2G-glucosylrutinoside)	[M-2H]-	Anthocyanidin-3-O-glycosides	149,875.89	111,082.76	410,511.32
4.774	609.14484	Rutin	[M-H]-	Flavonoid-3-O-glycosides	19,562,386.73	20,491,546.20	5,647,990.34
4.832	403.12411	Methyl (1S,4aR,7aR)-4a-hydroxy-7-(hydroxymethyl)-1-[(2S,3R,4S,5S,6R)-...]	[M-H]-	Iridoid O-glycosides	1,221,971.60	341,070.08	441,144.13
5.320	463.08640	Hyperoside	[M-H]-	Flavonoid-3-O-glycosides	4,079,385.39	2,252,446.51	4,689,667.40
5.424	593.14923	Datiscetin-3-O-rutinoside	[M-H]-	Flavonoid-3-O-glycosides	6,521,248.56	63,524.28	1,780,425.40
5.619	357.11853	Sweroside	[M-H]-	O-glycosyl compounds	42,129.06	2,149.74	22,213.32
5.727	623.19812	[(2R,3R,4S,5R,6R)-6-[2-(3,4-dihydroxyphenyl)ethoxy]-...]	[M-H]-	Coumaric acids and derivatives	35,673.66	48,072.48	26,435.90

5.936	515.11786	3,4-di-O-caffeoylquinic acid	[M-H]-	Quinic acids and derivatives	793,622.84	179,403.32	481,613.95
6.135	427.16052	(2R,3R,4R,5R,6S)-2-[[...]]	[M-H]-	Phenolic glycosides	9,813.34	17,324.43	98,100.00
6.733	312.12296	Feruloyltyramine	[M-H]-	Hydroxycinnamic acids and derivatives	0.00	2,826.19	0.00
6.942	301.03448	Quercetin	[M-H]-	Flavonols	566,574.15	310,455.20	1,789,256.01
6.944	285.04053	Kaempferol	[M-H]-	Flavonols	26,491.55	101,867.97	108,190.73
7.434	955.48907	3a-[(2S,3R,4S,5R,6R)-3,5-dihydroxy-6-[[...]]	[M-H]-	Triterpene saponins	3,825,344.11	1,316,469.75	1,702,889.89
7.640	281.13870	5,9-dihydroxy-7-(hydroxymethyl)-...	[M-H]-	Terpene lactones	3,358.10	27,375.99	9,165.91
7.668	269.04425	Galangin	[M-H]-	Flavonols	3,167.63	22,418.02	22,838.91
8.408	809.43079	(2S,3S,4S,5R,6R)-6-[[...]]	[M-H]-	Triterpene saponins	866,321.61	7,884,989.98	23,518,531.85
8.870	299.05576	Tectorigenin	[M-H]-	Isoflavones	99,041.20	67,142.14	70,846.91
9.180	793.43640	(1S,2R,4aS,6aR,6bR,10S,12aR)-10-[(2R,3R,4S,5R,6S)-...]	[M-H]-	Triterpene saponins	3,675,866.12	7,601,674.72	21,042,394.74
9.711	503.33636	Madecassic acid	[M-H]-	Triterpenoids	2,695,211.91	559,985.23	2,192,952.63
12.158	293.17834	Tetradecylsulfate	[M-H]-	Sulfuric acid monoesters	54,837.07	76,477.42	31,415.18
12.771	325.18341	Dodecylbenzenesulfonic acid	[M-H]-	Benzenesulfonic acids and derivatives	2,036,675.56	1,460,017.07	1,381,951.40
13.257	321.22012	Bis(2-ethylhexyl)phosphate	[M-H]-	Dialkyl phosphates	11,507.73	70,579.26	51,729.77
13.695	355.15472	Rutamarin	[M-H]-	Psoralens	0.00	8,440.44	7,720.44
14.119	387.18027	(4E,8E)-10-(4-hydroxy-6-methoxy-...)	[M-H]-	Terpene lactones	0.00	126,966.80	14,933.93
14.241	271.22754	16-Hydroxyhexadecanoic acid	[M-H]-	Long-chain fatty acids	1,145,927.49	217,472.46	8,096,467.66
14.371	455.35202	Ursolic acid	[M-H]-	Triterpenoids	222,438.89	442,698.93	1,273,028.50

Table S6 Differential metabolites between kratom and allied *Mitragyna* species based on fold change (FC), log_2 fold change (Log2FC), and variable importance in projection (VIP) scores derived from PLS-DA.

Metabolite name	Mean kratom	Mean allied *Mitragyna* species	FC*	Log2FC	VIP score	Higher in
7-hydroxymitragynine	6,269,339.28	63,737,228.78	0.10	-3.35	1.01	allied species
Brucine	13,967,909.33	121,017,594.93	0.12	-3.12	0.76	allied species
Corynoxine	ND	38,345,401.44	0.01	-6.62	0.84	allied species
Datiscetin-3-O-rutinoside	8,410,613.64	2,788,399.41	3.02	1.59	1.35	kratom
Gardneramine	ND	16,818,235.34	0.00	-8.03	0.84	allied species
Hyperoside	3,711,499.90	3,673,833.10	1.01	0.01	0.03	kratom
Mitragynine	21,754,867.64	ND	14.85	3.89	1.03	kratom
Procyanidin B1	4,251,579.79	ND	3.52	1.81	1.66	kratom
Rutin	21,020,383.49	15,233,974.42	1.38	0.46	0.61	kratom

Note: *Fold change (FC) was calculated based on the comparison between kratom and allied *Mitragyna* species, ND: not detect

Table S7 List of DNA barcoding primers used in this study

DNA Barcoding regions	Primer names	Sequence (5' > 3')	References
ITS	ITS1	CCTTATCATTTAGAGGAAGGAG	[1]
	ITS2	TCCTCCGCTTATTGATATGC	[2]
*mat*K	matK-1RKIM-f	ACCCAGTCCATCTGGAAATCTTGGTTC	[3]
	matK-3RKIM-r	CGTACAGTACTTTTGTGTTTACGAG	[3]
*rbc*L	rbcL1F	ATGTCACCACAAACAGAGACTAAAGC	[4]
	rbcL724R	GTAAAATCAAGTCCACCGCG	[4]
*trn*H-*psb*A	trnH (GUG)	CGTAACAAGGTTTCCGTAGGTGAA	[5]
	psbA	GTTATGCATGAACGTAATGCTC	[5]
*trn*L-F	trnL-Fc′	CGAAATCGGTAGACGCTACG	[6]
	trnL-Ff′	ATTTGAACTGGTGACACGAG	[6]
*rpo*C1	rpoC1-F	GGCAAAGAGGGAAGATTTCG	[7]
	rpoC1-R	CCATAAGCATATCTTGAGTTGG	[7]
*ycf*1	ycf1bF	TCTCGACGAAAATCAGATTGTTGTGAAT	[8]
	ycf1bR	ATACATGTCAAAGTGATGGAAAA	[8]
SLS2	SLS2-F	CAGAGAGAACAGGCAGACCA	[9]
	SLS2-R	TTACTGGCTTCCCCTGCTTG	[9]

1. Stanford, A.M.; Harden, R.; Parks, C.R. Phylogeny and biogeography of Juglans (Juglandaceae) based on matK and ITS sequence data. *American Journal of Botany* **2000**, 87, 872-882.
2. White, T.J.; Bruns, T.; Lee, S.; Taylor, J. Amplification and direct sequencing of fungal ribosomal RNA genes for phylogenetics. *PCR protocols: a guide to methods and applications* **1990**, 18, 315-322.
3. MB, H. Four primer pairs for the amplification of chloroplast intergenic regions with intraspecific variation. *Mol Ecol* **1999**, 8, 521-522.
4. Fay, M.F.; Bayer, C.; Alverson, W.S.; de Bruijn, A.Y.; Chase, M.W. Plastid rbcL sequence data indicate a close affinity between Diegodendron and Bixa. *Taxon* **1998**, 47, 43-50.
5. Kuzmina, M.L.; Johnson, K.L.; Barron, H.R.; Hebert, P.D. Identification of the vascular plants of Churchill, Manitoba, using a DNA barcode library. *BMC ecology* **2012**, 12, 1-11.
6. Taberlet, P.; Gielly, L.; Pautou, G.; Bouvet, J. Universal primers for amplification of three non-coding regions of chloroplast DNA. *Plant Mol Biol* **1991** 17, 1105–1109
7. Hollingsworth, P.M.; Forrest, L.L.; Spouge, J.L.; Hajibabaei, M. A DNA barcode for land plants. *Proc Nat Acad Sci USA* **2009** 106, 12794–12797
8. Dong, W.; Xu, C.; Li, C.; Sun, J.; Zuo, Y.; Shi, S.; Cheng, T.; Guo, J.; Zhou, S. ycf1, the most promising plastid DNA bar- code of land plants. *Sci Rep* **2015** 5, 8348.
9. Cowan, A.F.; Elkins, K.M. Detection and identification of kratom (*Mitragyna speciosa*) and marijuana (*Cannabis sativa*) by a real-time polymerase chain reaction high-resolution melt duplex assay. *Journal of Forensic Sciences* **2020**, 65, 52-60.

Sample	UV Spectrum (Mitragynine)	UV purity (%)
Kratom: Kan Daeng	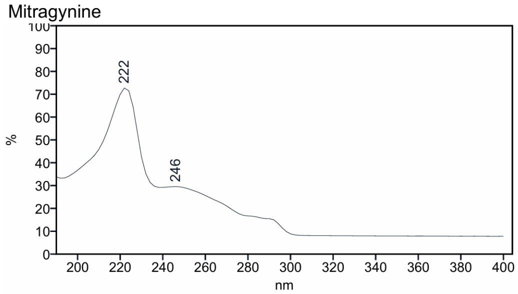	99.88
Kratom: Hang Kang	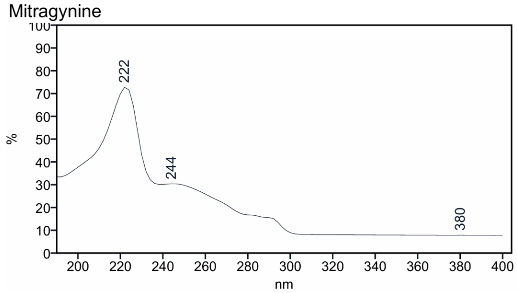	99.95
Kratom: Kan Khiao	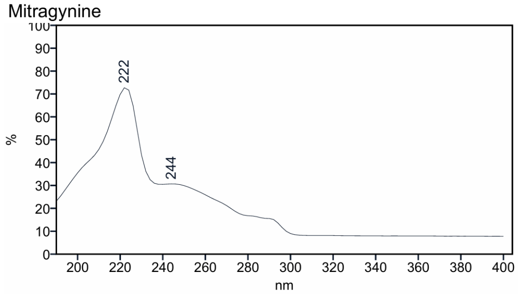	99.99
Standard Mitragynine	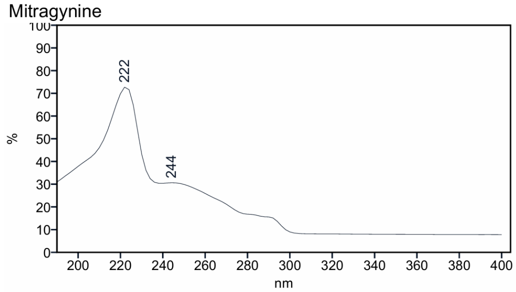	100.00

Figure S1 The identity of mitragynine was confirmed by its UV spectrum and UV purity (%).

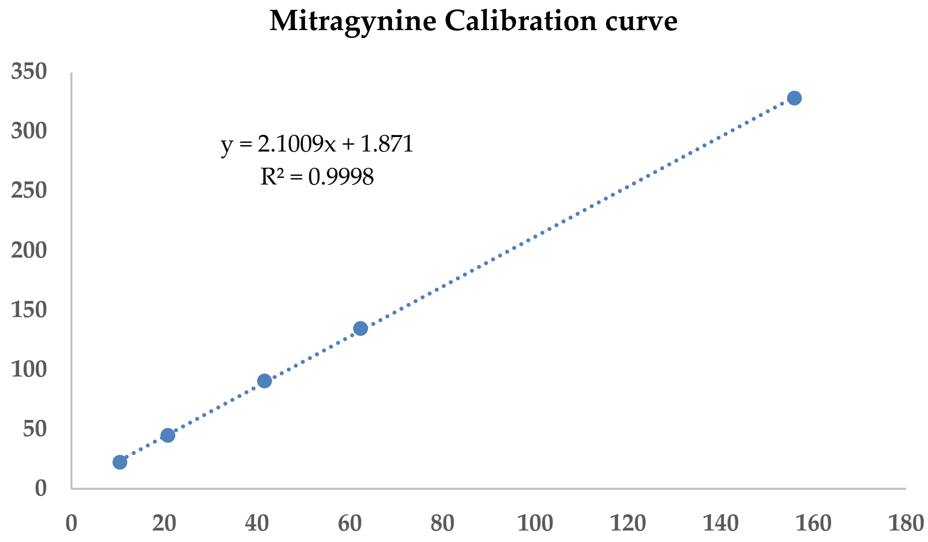

Figure S2 Calibration curve of standard mitragynine showing linear regression (y = 2.1009x + 1.871) with a coefficient of determination (R^2 = 0.9998)

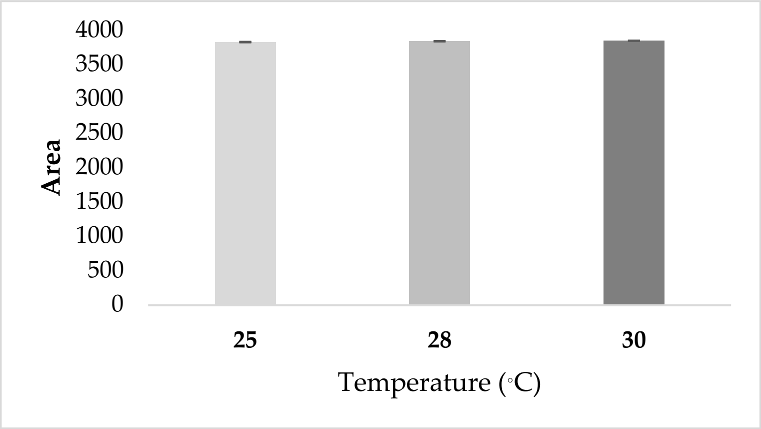

Figure S3 Robustness evaluation of the UHPLC method showing the effect of column temperature variation (25, 28, and 30 °C) on the peak area of mitragynine
